# Sex-dependent associations of plasma high-density lipoprotein cholesterol and mortality risk in healthy older men and women: two prospective cohort studies

**DOI:** 10.1007/s11357-023-00904-4

**Published:** 2023-08-23

**Authors:** Sultana Monira Hussain, Andrew M Tonkin, Gerald F Watts, Paul Lacaze, Chenglong Yu, Lawrence J Beilin, Zhen Zhou, Anne B Newman, Johannes T Neumann, Cammie Tran, John J McNeil

**Affiliations:** 1 School of Public Health and Preventive Medicine, Monash University, Melbourne, Victoria Australia; 2 Department of Medical Education, Melbourne Medical School, The University of Melbourne, Parkville, Victoria Australia; 3 School of Medicine, University of Western Australia, Perth, Australia; 4 Menzies Institute for Medical Research, University of Tasmania, Hobart, Australia; 5 Center for Aging and Population Health, University of Pittsburgh, Pittsburgh, USA; 6 Department of Cardiology, University Heart & Vascular Center (UHZ), Hamburg, Germany; 7 German Center for Cardiovascular Research (DZHK), Partner Site Hamburg/Kiel/Lübeck, Hamburg, Germany

**Keywords:** High-density lipoprotein cholesterol, All-cause mortality, Cardiovascular disease mortality, Cancer mortality, “Non-cancer non-cardiovascular” mortality, Older adults

## Abstract

The relationship between high plasma high-density lipoprotein cholesterol (HDL-C) and cause and mortality are not well established in healthy older people. This study examined the associations between HDL-C levels and mortality in initially healthy older men and women. This analysis included participants from the Aspirin in Reducing Events in the Elderly (ASPREE; *n*=18,668) trial and a matched cohort from the UK Biobank (UKB; *n*=62,849 ≥65 years). Cox regression was used to examine hazard ratios between HDL-C categories <1.03 mmol/L, 1.03–1.55 mmol/L (referent category), 1.55–2.07 mmol/L, and >2.07 mmol/L and all-cause, cancer, cardiovascular disease (CVD), and “non-cancer non-CVD” mortality. Genetic contributions were assessed using a polygenic score for HDL-C. Among ASPREE participants (aged 75±5 years), 1836 deaths occurred over a mean follow-up of 6.3±1.8 years. In men, the highest category of HDL-C levels was associated with increased risk of all-cause (HR 1.60, 95% CI 1.26–2.03), cancer (HR 1.37, 95% CI 0.96–2.00), and “non-cancer non-CVD” mortality (HR 2.35, 95% CI 1.41–3.42) but not CVD mortality (HR 1.08, 95% CI 0.60–1.94). The associations were replicated among UKB participants (aged 66.9±1.5 years), including 8739 deaths over a mean follow-up of 12.7±0.8 years. There was a non-linear association between HDL-C levels and all-cause and cause-specific mortality. The association between HDL-C levels and mortality was unrelated to variations in the HDL-C polygenic score. No significant association was found between HDL-C levels and mortality in women. Higher HDL-C levels are associated with increased risk from cancer and “non-cancer non-CVD” mortality in healthy older men but no such relationship was observed in women.

**Supplementary Information:**

The online version contains supplementary material available at 10.1007/s11357-023-00904-4.

## Introduction

High-density lipoprotein cholesterol (HDL-C) particles are complexes of lipids and apolipoproteins that transport cholesterol in the circulation through a process known as “reverse cholesterol transport” [1]. Higher concentrations of HDL-C in plasma are typically seen as protective markers for cardiovascular disease (CVD) risk. However, recent observational studies have reported that very high HDL-C levels are associated with increased risks of all-cause mortality and various chronic conditions [2–13].

The HDL-C system has a range of potential physiological functions that could explain its relationship to a diverse range of chronic diseases [14]. Proteins embedded in the phospholipid coating of HDL-C may be related to coagulation, immune function, and inflammation [14]. A study of genetic variants associated with HDL-C has suggested an increased risk of mortality from sepsis [15]. The specific pathophysiology underlying an increase in mortality is unclear. Various aspects of HDL’s biological function may change with increasing age [16, 17]. Furthermore, women tend to have higher HDL-C levels compared to men; however, after menopause, the sex difference in HDL-C levels tends to narrow [18]. On the contrary, some studies have reported no changes, while others have even observed an increase in HDL-C after menopause [19].

There is little information on the sex-specific relationship between high HDL-C and all-cause and cause-specific mortality in older populations, with a single report from the US Health and Retirement Study (HRS) [6] reporting an increased cardiovascular and all-cause mortality among participants exclusively from this age group. The availability of data from the Aspirin in Reducing Events in the Elderly (ASPREE) clinical trial and the UK Biobank (UKB) has provided the opportunity to comprehensively explore the relationship between high HDL-C levels and cause-specific and sex-specific mortality in older populations.

## Methods

### Study settings

We analyzed data from the ASPREE trial and ASPREE-XT (ASPREE–eXTension) period, an ongoing, follow-up of the study, and a subset of the participants of the UKB as a validation cohort. ASPREE was approved by the Monash University Human Research Ethics Committee and other allied institution ethics committees. UKB was approved by the North West Multi-Centre Research ethics committee in the UK. Analysis of the UKB was approved under project ID 47061. All participants from both the studies provided written informed consent. This study followed the Strengthening the Reporting of Observational Studies in Epidemiology (STROBE) reporting guidelines.

### ASPREE

#### Study participants

The ASPREE trial recruited 19,114 community-dwelling adults from Australia (aged ≥70 years) and the USA (aged ≥65 years). Details of participant recruitment and primary outcomes have been reported previously [20, 21]. Briefly, participants were recruited from March 2010 to December 2014 and were randomized to receive 100 mg aspirin daily or matching placebo [20–22]. Participants were excluded if they had evident CVD events, dementia, physical disability, or a chronic illness expected to limit survival to <5 years. Individuals with uncontrolled high blood pressure (SBP ≥180 mmHg and/or DBP ≥105 mmHg) were also excluded.

#### Standard assessment

Data were collected from annual in-person visits and 6-monthly telephone contacts. At these visits, data regarding medical comorbidities and other related health parameters were collected [21].

#### Measurement of serum cholesterol

Fasting lipids, including HDL-C and non-HDL-C, were measured by local pathology providers from fresh plasma [20, 21].

#### Assessment of mortality

Mortality events were identified during the routine trial activity and afterwards as part of an ongoing follow-up. Mortality events were confirmed by reviewing of health records [22] and were confirmed from two independent sources and by linkage to the National Death Index in the relevant country. Cause of death was established by examining the progression of the final illness or incident and was considered to be the single disease most likely to have initiated the trajectory toward death.

### UK Biobank

Study participants. UKB is a long-term biomedical study containing in-depth genetic and health information from half a million individuals from the UK general population recruited between 2006 and 2010. Participants who were ≥65 years of age were included in this analysis. Those with a record of diagnosed dementia or CVD events earlier than at first enrolment were identified using records of ICD-10 codes and were excluded (Supplementary Method), as were those with SBP ≥180 mmHg and/or DBP ≥105 mmHg at enrolment.

#### Standard assessments

Sociodemographic data including age, sex, ethnicity, body mass index (BMI), blood pressure, estimated glomerular filtration rate (eGFR), diabetes, smoking, and alcohol use were obtained at recruitment.

#### Measurement of plasma cholesterol

Plasma cholesterol including HDL-C was assayed with the AU5800 analytical platform (Beckman Coulter).

#### Assessment of mortality

Cause of death for each participant has been identified by the ICD-10 code. The database is linked to Hospital Episode Statistics data and to national death registries which send death notifications to UKB on a regular basis. The codes for cause-specific mortality are detailed in Supplementary Method.

### Polygenic risk score for HDL-C (ASPREE and UKB)

In participants where genotype data was available for both ASPREE and UKB, the polygenic risk scores (PRS) were generated using 309 independent genome-wide significant variants (*P*<5×10^−8^) associated with HDL-C (Table S1). These genomic variants were sourced from a large-scale genome-wide association study (GWAS) [23] based on ~300,000 multi-ethnic participants in the Million Veteran Program and performed using GCTA-COJO software [24]. The PRS was calculated by summing all these variants weighted by reported beta coefficients in the GWAS and then converted to *Z* scores [11]. The distribution of the PRS *Z* score is presented in Fig. S1.

### Statistical analysis

Analysis was performed from October 2022 to February 2023. Cox regression was used to calculate the hazard ratios (HR) and 95% confidence interval (CI) between HDL-C categories (<1.03 mmol/L [<40 mg/dL], 1.03–1.55 mmol/L [40–60mg/dL], 1.55–2.07 mmol/L [60–80mg/dL], and >2.07 mmol/L [>80 mg/dL]) and all-cause mortality and a competing risk analysis (Fine-Gray) was performed for cancer, CVD, and “non-cancer non-CVD” specific mortality that consider any specific cause of death as a competing event using 1.55–2.07 mmol/L [40–60 mg/dL] as reference category. Various analytical models were adjusted for one or more of age, country of birth/ethnic background, BMI, physical activity (walking outside <30 min vs. >30 min), alcohol use, smoking status, hypertension, diabetes, chronic kidney disease (eGFR <60 mL/min), and non-HDL-C. There was no interaction between HDL-C levels and age, HDL-C and dyslipidemia, and HDL-C and physical activity for mortality events.

The associations between HDL-C and all-cause mortality and cause-specific mortality were examined using restricted cubic spline curves that permitted non-linear associations, treating an HDL-C level of 1.42 mmol/L [55 mg/dL] (median in ASPREE population) as the reference with aforementioned covariate adjustment.

To examine whether HDL-C genetic variations contribute to the associations substantially, we analyzed data including the HDL-C PRS *z* score and 10 principal components (PC) for population structure for a subgroup of ASPREE and a subgroup of UKB population for whom genetic data were available.

Sensitivity analyses were performed excluding the US population, restricting the analysis to those who undertook minimal physical activity (walking outside <30 minutes/day), excluding those with weight loss of over 5% in the first two years after recruitment, excluding men who reported a history of prostate cancer or were taking antiandrogen or any testosterone therapy or DHEA for ASPREE, excluding women who reported taking any hormone replacement therapy. For UKB, we restricted our analyses to Caucasians and who walked outside <30 minutes/day.

All analyses were performed using Stata MP version 17 for Windows (StataCorp LP, College Station, TX).

## Results

### ASPREE analysis

Of the 19,114 individuals enrolled into ASPREE, 18,668 with plasma HDL-C concentration measured at baseline were included. There were 43.5% men and 56.5% women. During an average of 6.3 (SD 1.8) years of follow-up, there were 1836 mortality events. Participants within the highest category of HDL-C levels constituted 5.9% of men and 21.2% of women. Men and women in the highest HDL-C category, compared with the reference category, on average had lower BMI, were less likely to be current/former smokers, were more likely to be physically active, and had a lower prevalence of hypertension, diabetes, and chronic kidney disease (Table 1). Participants in this category also had lower levels of non-HDL-C and were less likely to be on lipid-lowering medications. The range of HDL-C concentrations in this community-based healthy population was wider for women compared with men, and on average, women had higher HDL cholesterol (Fig. S2).

**Table 1:** Baseline characteristics of participants: overall and high-density lipoprotein cholesterol (HDL-C) category in the Aspirin in Reducing Events in the Elderly (ASPREE) cohort

	Overall	<40 mg/dL <1.03 mmol/L	40–60 mg/dL 1.03–1.55 mmol/L	60–80 mg/dL 1.55–2.07 mmol/L	>80 mg/dL >2.07 mmol/L	*P* value
*Male*						
*N* (%)	8126 (100)	1378 (17.0)	4359 (53.6)	1913 (23.5)	476 (5.9)	
Age, mean (SD), y	74.9 (4.5)	74.7 (4.3)	74.9 (4.5)	75.1 (4.5)	75.5 (4.6)	0.13
Country of birth						0.002
Australia	7319 (90.1)	1208 (87.7)	3925 (90.0)	1745 (91.2)	441 (92.7)	
USA	807 (9.9)	170 (12.3)	434 (10.0)	168 (8.8)	35 (7.4)	
Low activity (walked outside <30 minutes), *n* (%)	2787 (34.3)	558 (40.5)	1508 (34.6)	602 (31.5)	119 (25.0)	<0.001
BMI, mean (SD) (kg/m^2^)	28.0 (3.9)	29.5 (4.1)	28.2 (3.8)	26.9 (3.7)	25.7 (3.5)	<0.001
Waist circumference, mean (SD), cm	102.0 (10.8)	106.2 (10.7)	102.7 (10.4)	98.9 (10.5)	96.0 (10.3)	<0.001
Current/former smoking, *n* (%)	3506 (43.2)	615 (44.6)	1944 (44.6)	776 (40.6)	171 (35.9)	<0.001
Current alcohol use, *n* (%)	6757 (83.2)	1013 (73.5)	3612 (82.9)	1691 (88.4)	441 (92.7)	<0.001
Education, *n* (%)						<0.001
<12 years of schooling	4492 (55.3)	832 (60.4)	2396 (55.0)	1018 (53.2)	246 (51.7)	
>12 years of schooling	3634 (44.7)	546 (39.6)	1963 (45.0)	895 (46.8)	230 (48.3)	
Hypertension, *n* (%)	6121 (75.3)	1097 (79.6)	3228 (74.1)	1440 (75.3)	356 (74.8)	0.001
Systolic blood pressure, mean (SD), mm/Hg	141.1 (15.9)	140.2 (15.7)	140.7 (15.7)	142.3 (16.0)	143.0 (16.8)	<0.001
Diastolic blood pressure, mean (SD), mm/Hg	78.1 (9.6)	78.0 (10.0)	78.2 (9.5)	78.1 (9.6)	77.9 (9.7)	0.87
Mean arterial pressure, mean (SD), mm/Hg	99.1 (10.3)	98.7 (10.6)	99.0 (10.2)	99.5 (10.4)	99.6 (10.9)	0.10
Chronic kidney disease (eGFR < 60 mL/min) (%) (*n*=7949)	1339/7949 (16.8)	331/1343 (24.7)	724/4263 (17.0)	230/1878 (12.3)	54/465 (11.6)	<0.001
Diabetes, *n* (%)	1013 (12.5)	288 (20.9)	545 (12.5)	148 (7.7)	32 (6.7)	<0.001
Prefrail/frail, *n* (%)	3187 (39.2)	593 (43.0)	1715 (39.3)	701 (36.6)	178 (37.4)	0.002
On trial medication, *n* (%)	4057 (49.3)	690 (50.1)	2163 (49.6)	962 (50.3)	242 (50.8)	0.93
HDL-C PRS, mean (SD), *z* score (*n*=5885)	0.026 (1.00)	−0.576 (0.96)	−0.011 (0.93)	0.364 (0.97)	0.72 (0.93)	<0.001
Total cholesterol (mmol/L)	5.0 (0.9)	4.6 (1.0)	5.0 (0.9)	5.3 (0.9)	5.5 (0.8)	<0.001
Non-HDL-C (mmol/L)	3.6 (0.9)	3.7 (0.9)	3.7 (0.8)	3.5 (0.9)	3.1 (0.8)	<0.001
Low-density lipoprotein (mmol/L)	3.0 (0.8)	2.9 (0.9)	3.1 (0.8)	3.0 (0.8)	2.7 (0.8)	<0.001
Triglyceride (mmol/L)	1.3 (0.7)	1.9 (1.0)	1.3 (0.6)	1.0 (0.5)	0.8 (0.3)	<0.001
Total cholesterol/high-density lipoprotein (mmol/L)	3.8 (1.1)	5.1 (1.2)	3.9 (0.8)	3.0 (0.6)	2.3 (0.4)	<0.001
Lipid-lowering medications (%) (*n*=7630)	2429/7630 (31.8)	459/1312 (35.0)	1296/4087 (31.7)	543/1789 (30.4)	131/442 (29.6)	0.06
Hydrophilic, *n* (%)	708 (9.3)	149 (11.4)	352 (8.6)	165 (9.2)	42 (9.5)	
Lipolethic, *n* (%)	1565 (20.5)	276 (21.0)	857 (21.0)	350 (19.6)	82 (18.6)	
*Female*						
*N* (%)	10,542 (100)	392 (3.7)	3732 (35.4)	4185 (39.7)	2233 (21.2)	
Age, mean (SD), y	75.2 (4.6)	74.9 (4.5)	75.0 (4.6)	75.2 (4.6)	75.5 (4.6)	<0.001
Country of birth						<0.001
Australia	8945 (84.9)	321 (81.9)	3080 (82.5)	3587 (85.7)	1957 (87.6)	
USA	1597 (15.2)	71 (18.1)	652 (17.5)	598 (14.3)	376 (12.4)	
Low activity (walked outside <30 minutes), *n* (%)	4413 (41.9)	217 (55.4)	1730 (46.4)	1661 (39.7)	805 (36.1)	<0.001
BMI (kg/m^2^)	28.2 (5.2)	30.6 (5.1)	29.8 (5.4)	27.7 (4.8)	26.0 (4.7)	<0.001
Waist circumference, mean (SD), cm	93.3 (13.0)	100.7 (11.9)	97.5 (12.7)	92.0 (12.2)	87.5 (12.2)	<0.001
Current/former smoking, *n* (%)	3706 (35.2)	130 (33.2)	1296 (34.7)	1425 (34.1)	855 (38.3)	0.005
Current alcohol use, *n* (%)	7535 (71.5)	227 (57.9)	2416 (64.7)	3099 (74.1)	1793 (80.3)	<0.001
Education, *n* (%)						<0.001
<12 years of schooling	6195 (58.8)	267 (68.1)	2275 (61.0)	2443 (58.4)	1210 (54.2)	
>12 years of schooling	4346 (41.2)	125 (31.9)	1457 (39.0)	1742 (41.6)	1022 (45.8)	
Hypertension, *n* (%)	7738 (73.4)	335 (85.5)	2897 (77.6)	2967 (70.9)	1539 (68.9)	<0.001
Systolic blood pressure, mean (SD), mm/Hg	137.6 (16.8)	137.0 (16.6)	137.9 (16.5)	137.4 (17.1)	137.8 (16.9)	0.39
Diastolic blood pressure, mean (SD), mm/Hg	76.6 (10.2)	76.0 (10.4)	76.9 (10.3)	76.5 (10.2)	76.6 (10.2)	0.16
Mean arterial pressure, mean (SD), mm/Hg	97.0 (11.0)	96.2 (10.9)	97.2 (10.7)	96.8 (11.0)	97.0 (11.0)	0.17
Chronic kidney disease (eGFR <60 mL/min) (%) (*n*=10,350)	2006/10,350 (19.4)	114/383 (29.8)	820/3674 (22.3)	719/4100 (17.5)	353/2193 (16.1)	<0.001
Diabetes, *n* (%)	975 (9.3)	111 (28.3)	495 (13.3)	262 (6.3)	107 (4.8)	<0.001
Prefrail/frail, *n* (%)	4495 (42.6)	190 (48.5)	1650 (44.2)	1733 (41.4)	922 (41.3)	0.004
Hormone replacement therapy, *n* (%)	894 (8.5)	38 (9.7)	307 (8.2)	337 (8.1)	212 (9.5)	0.17
On trial medication, *n* (%)	5251 (49.8)	198 (50.5)	1852 (49.6)	2107 (50.4)	1094 (49.0)	0.75
HDL-C PRS, mean (SD), *z* score	−0.019 (1.00)	−0.726 (0.95)	−0.392 (0.94)	0.085 (0.93)	0.847 (0.94)	<0.001
Total cholesterol (mmol/L)	5.4 (1.0)	4.7 (1.1)	5.2 (1.0)	5.5 (0.9)	5.8 (0.9)	<0.001
Non-HDL-C (mmol/L)	3.7 (1.0)	3.8 (1.1)	3.9 (1.0)	3.7 (0.9)	3.4 (0.9)	<0.001
Low-density lipoprotein (mmol/L)	3.1 (0.9)	2.8 (0.9)	3.1 (0.9)	3.2 (0.9)	3.0 (0.8)	<0.001
Triglyceride (mmol/L)	1.3 (0.6)	2.1 (1.0)	1.6 (0.6)	1.2 (0.5)	0.9 (0.4)	<0.001
Total cholesterol/high-density lipoprotein (mmol/L)	3.3 (1.1)	5.2 (3.1)	3.9 (0.8)	3.1 (0.6)	2.5 (0.4)	<0.001
Lipid-lowering medications (%) (*n*=10,184)	3882/10,184 (38.1)	186/390 (47.7)	1546/3635 (42.5)	1452/4036 (36.0)	698/2123 (32.9)	<0.001
Hydrophilic, *n* (%)	1105 (10.9)	55 (14.1)	433 (11.9)	419 (10.4)	198 (9.3)	
Lipolethic, *n* (%)	2455 (24.1)	120 (30.8)	999 (27.5)	901 (22.3)	435 (20.5)	

#### HDL-C levels and mortality

Associations between HDL-C categories and all-cause and cause-specific mortality in men and women are presented in Table 2. In men, compared to the reference category, the rate of all-cause and cause-specific mortality was higher in the >2.07 mmol/L (80 mg/dL) HDL-C category (all-cause 182/10,000 person-years vs. 312/10,000 person-years; cancer 84/10,000 person-years vs. 122/10,000 person-years; CVD 46/10,000 person-years vs. 49/10,000 person-years; non-cancer non-CVD 51/10,000 person-years vs. 142/10,000 person-years).

**Table 2:** Association between baseline HDL-C levels and all-cause and cause-specific mortality in males and females (HR, 95% CI), in the Aspirin in Reducing Events in the Elderly (ASPREE) cohort

	HDL levels							
	<40 mg/dL <1.03 mmol/L	40–60 mg/dL 1.03–1.55 mmol/L	60–80 mg/dL 1.55–2.07 mmol/L	>80 mg dL >2.07 mmol/L	<40 mg/dL <1.03 mmol/L	40–60 mg/dL 1.03–1.55 mmol/L	60–80 mg/dL 1.55–2.07 mmol/L	>80 mg dL >2.07 mmol/L
	Male				Female			
All-cause								
*N* (%)	175/1387 (12.7)	498/4359 (11.4)	222/1913 (11.6)	90/476 (18.9)	34/392 (8.7)	304/3732 (8.2)	334/4185 (8.0)	179/2233 (8.0)
Rate/10,000 person-years	203	182	184	312	138	129	125	125
Model 1	1.16 (0.98–1.38)	1	0.99 (0.85–1.16)	1.64 (1.31–2.05)	1.13 (0.79–1.61)	1	0.95 (0.81–1.10)	0.92 (0.77–1.11)
Model 2	1.12 (0.94–1.33)	1	0.99 (0.85–1.17)	1.62 (1.29–2.05)	1.06 (0.74–1.51)	1	0.99 (0.84–1.16)	0.97 (0.80–1.17)
Model 3	1.05 (0.88–1.26)	1	1.01 (0.86–1.19)	1.60 (1.26–2.03)	1.03 (0.72–1.47)	1	1.01 (0.86–1.18)	0.96 (0.79–1.17)
Cancer								
*N* (%)	88/1291 (6.8)	231/4092 (5.7)	104/1795 (5.8)	35/421 (8.3)	14/372 (3.8)	152/3580 (4.3)	146/3997 (3.7)	68/2122 (3.2)
Rate/10,000 person-years	102	84	86	122	57	64	55	48
Model 1	1.24 (0.97–1.59)	1	1.01 (0.80–1.27)	1.39 (0.99–2.00)	0.91 (0.52–1.56)	1	0.83 (0.66–1.05)	0.71 (0.54–0.95)
Model 2	1.20 (0.94–1.54)	1	1.02 (0.81–1.29)	1.40 (0.98–2.01)	0.87 (0.50–1.50)	1	0.87 (0.69–1.09)	0.77 (0.57–1.03)
Model 3	1.12 (0.87–1.44)	1	1.03 (0.81–1.30)	1.37 (0.96–2.00)	0.85 (0.49–1.47)	1	0.88 (0.70–1.12)	0.73 (0.53–0.99)
CVD								
*N* (%)	40/1243 (3.2)	127/3988 (3.2)	42/1733 (2.4)	14/400 (3.5)	10/368 (2.7)	67/3495 (1.9)	80/3931 (2.0)	55/2109 (2.6)
Rate/10,000 person-years	46	46	35	49	41	28	29	39
Model 1	0.93 (0.64–1.50)	1	0.73 (0.52–1.04)	1.00 (0.57–1.73)	1.58 (0.81–3.07)	1	1.02 (0.74–1.41)	1.27 (0.89–1.81)
Model 2	0.99 (0.69–1.42)	1	0.74 (0.52–1.06)	0.97 (0.55–1.73)	1.41 (0.73–2.75)	1	1.11 (0.80–1.54)	1.40 (0.97–2.03)
Model 3	0.93 (0.65–1.35)	1	0.79 (0.55–1.12)	1.08 (0.60–1.94)	1.38 (0.71–2.70)	1	1.16 (0.83–1.63)	1.48 (1.00–2.18)
Non-cancer non-CVD								
*N* (%)	47/1250 (3.8)	140/4001 (3.5)	76/1767 (4.3)	41/427 (9.6)	10/368 (2.7)	85/3513 (2.4)	108/3959 (2.7)	56/2110 (2.7)
Rate/10,000 person-years	54	51	63	142	41	36	40	39
Model 1	1.13 (0.81–1.57)	1	1.20 (0.91–1.59)	2.62 (1.85–3.71)	1.21 (0.63–2.33)	1	1.09 (0.82–1.44)	1.02 (0.73–1.43)
Model 2	1.09 (0.78–1.53)	1	1.17 (0.87–1.55)	2.52 (1.76–3.62)	1.14 (0.59–2.19)	1	1.12 (0.84–1.50)	0.98 (0.69–1.39)
Model 3	1.04 (0.74–1.46)	1	1.19 (0.89–1.58)	2.35 (1.41–3.42)	1.09 (0.56–2.11)	1	1.13 (0.84–1.52)	0.99 (0.69–1.42)

Model 1: adjusted for age. Model 2: adjusted for age, country of birth, BMI, physical activity, alcohol use, smoking status, level of education, 100 mg aspirin. Model 3: adjusted for age, country of birth, BMI, physical activity, alcohol use, smoking status, level of education, 100 mg aspirin, non-HDL-C, hypertension, diabetes, chronic kidney disease

Compared with men in the reference category, men with >2.07 mmol/L (>80 mg/dL) HDL-C were at increased risk of all-cause (HR 1.60, 95% CI 1.26–2.03), cancer (HR 1.37, 95% CI 0.96–2.00), and “non-cancer non-CVD” (HR 2.35, 95% CI 1.41–3.42) mortality. Hazard ratios were only modestly changed after adjustment for age, sex, country of birth, BMI, physical activity, alcohol use, smoking status, hypertension, diabetes, chronic kidney disease, and non-HDL-C. There was no association between the highest HDL-C category and CVD mortality in men (HR 1.08, 95% CI 0.60–1.94).

Among women, the rate of all-cause and cause-specific mortality did not increase in the highest HDL-C category compared with the reference category. No significant association between high HDL-C and all-cause or cause-specific mortality was observed in women.

#### Non-linearity

**Fig. 1:**
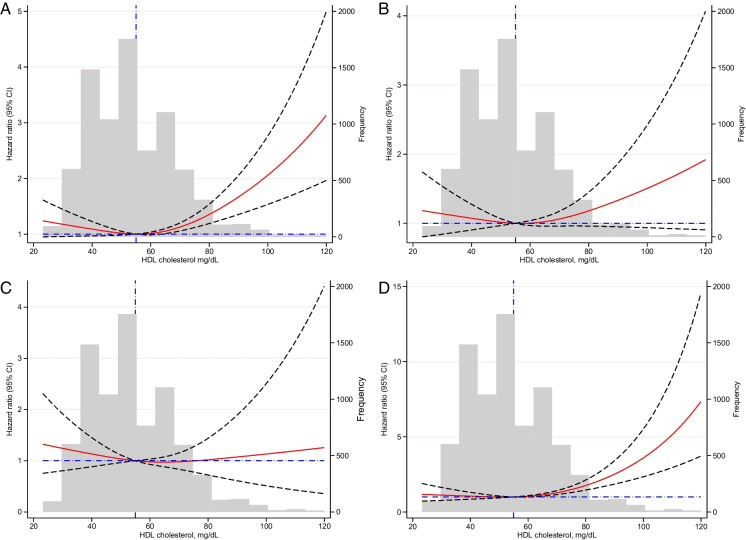
Non-linear association between high-density lipoprotein (HDL) cholesterol levels in men in the Aspirin in Reducing Events in the Elderly (ASPREE) cohort. **A** All-cause mortality. **B** Cancer mortality. **C** CVD mortality. **D** Non-cancer non-CVD mortality

**Fig. 2:**
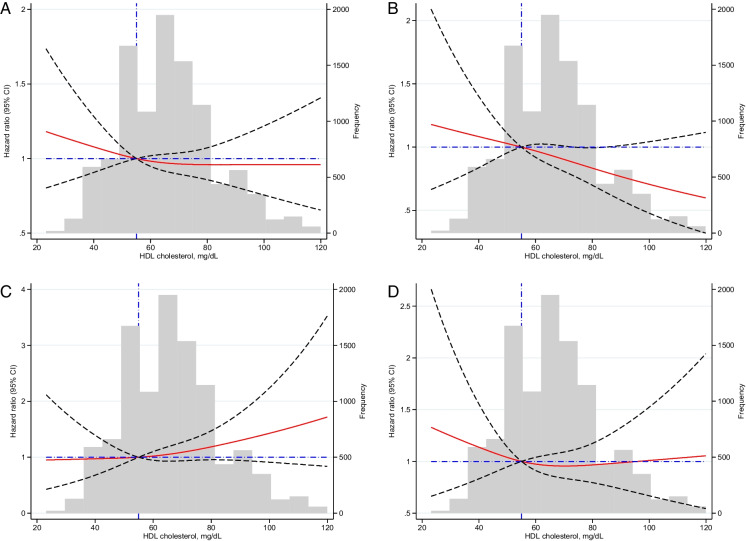
Non-linear association between high-density lipoprotein (HDL) cholesterol levels in women in the Aspirin in Reducing Events in the Elderly (ASPREE) cohort. **A** All-cause mortality. **B** Cancer mortality. **C** CVD mortality. **D** Non-cancer non-CVD mortality

Using adjusted restricted cubic spline curves, a non-linear association between HDL-C levels and risk of all-cause mortality, cancer mortality, and non-cancer non-CVD mortality was observed in men (Fig. 1). No such associations were observed in women (Fig. 2).

#### Sensitivity analyses

Results of the sensitivity analyses are shown in Tables S2–S4. Results remained unchanged for males and females, when the analyses were restricted for participants from Australia and participants who did not have unintentional weight loss. However, when women who were on hormone replacement therapy were excluded although high HDL-C levels were not associated with increased risk of all-cause, cancer, and “non-cancer non-CVD” mortality, there was a significant association between the highest category of HDL-C and CVD mortality (HR 1.63, 95% CI 1.09–2.40). Based on the few numbers of observations in CVD mortality category in these women, the result needs to be interpreted with caution.

### UK Biobank

After applying exclusion criteria, 62,849 participants remained for analysis. There were 47.3% men and 52.7% women. Among these participants, 8739 mortality events were recorded on an average of 12.7 (SD 0.8) years of follow-up. The distributions of HDL-C among men and women are presented in Fig. S3. As with ASPREE, the range of HDL-C concentrations in the UKB was wider in women than men, and on average, women had higher HDL-C.

There were 2.3% of men and 11.7% of women in the highest HDL-C category. Men and women in the highest HDL-C category compared to the reference category were more likely to be Caucasians, have lower BMI, were physically active, and were less likely to be current/former smokers. Participants with high HDL-C had a lower prevalence of diabetes and chronic kidney disease, but a similar prevalence of hypertension. Non-HDL-C levels were lower in those with HDL-C >2.07 mmol/L (>80 mg/dL) compared with the reference category (Table S5).

#### HDL-C levels and mortality

The associations between baseline HDL-C levels and all-cause and cause-specific mortality in men and women in the UKB are presented in Table 3. In men, compared to the reference category, the rates of all-cause and cause-specific mortality were higher within the high HDL-C category (all-cause 136/10,000 person-years vs. 172/10,000 person-years; cancer 65/10,000 person-years vs. 81/10,000 person-years; CVD 19/10,000 person-years vs. 22/10,000 person-years; non-cancer non-CVD 51/10,000 person-years vs. 69/10,000 person-years).

**Table 3:** Association between baseline high-density lipoprotein cholesterol (HDL-C) levels and all-cause and cause-specific mortality in males and females (HR, 95% CI), in the UK Biobank (UKB) cohort

	HDL levels							
	<40 mg/dL <1.03 mmol/L	40–60 mg/dL 1.03–1.55 mmol/L	60–80 mg/dL 1.55–2.07 mmol/L	>80 mg dL >2.07 mmol/L	<40 mg/dL <1.03 mmol/L	40–60 mg/dL 1.03–1.55 mmol/L	60–80 mg/dL 1.55–2.07 mmol/L	>80 mg dL >2.07 mmol/L
	Male				Female			
All-cause								
*N* (%)	1242/5861 (21.2)	2929/18,114 (16.2)	772/5084 (15.2)	134/675 (19.9)	213/1171 (18.2)	1692/14,733 (11.5)	1346/13,322 (10.1)	411/3888 (10.6)
Rate/10,000 person-years	181	136	127	172	152	94	83	87
Model 1	1.34 (1.25–1.43)	1	0.94 (0.87–1.02)	1.28 (1.08–1.53)	1.62 (1.40–1.87)	1	0.88 (0.82–0.95)	0.93 (0.83–1.04)
Model 2	1.23 (1.15–1.08)	1	1.00 (0.92–1.08)	1.42 (1.19–1.70)	1.44 (1.23–1.68)	1	0.91 (0.85–0.99)	1.00 (0.89–1.13)
Model 3	1.17 (1.08–1.25)	1	1.00 (0.92–1.09)	1.40 (1.18–1.67)	1.34 (1.15–1.57)	1	0.93 (0.86–1.00)	1.00 (0.89–1.12)
Cancer								
*N* (%)	632/5251 (12.0)	1411/16,596 (8.5)	372/4685 (7.9)	63 (10.4)	96/1054 (9.1)	918/13,959 (6.6)	736/12,712 (5.8)	229/3706 (6.2)
Rate/10,000 person-years	92	65	61	81	56	48	42	43
Model 1	1.41 (1.29–1.55)	1	0.94 (0.84–1.05)	1.25 (0.97–1.60)	1.35 (1.09–1.66)	1	0.88 (0.80–0.97)	0.95 (0.82–1.10)
Model 2	1.31 (1.18–1.44)	1	1.00 (0.88–1.12)	1.38 (1.07–1.79)	1.22 (0.98–1.53)	1	0.90 (0.82–1.00)	0.99 (0.85–1.16)
Model 3	1.26 (1.14–1.39)	1	1.00 (0.89–1.12)	1.36 (1.05–1.75)	1.20 (0.95–1.50)	1	0.91 (0.82–1.01)	0.99 (0.85–1.15)
CVD								
*N* (%)	180/4799 (3.8)	411/15,596 (2.6)	86/4399 (2.0)	17/558 (3.1)	39/997 (3.9)	167/13,208 (1.3)	103/12,079 (0.9)	32/3509 (0.9)
Rate/10,000 person-years	26	19	14	22	27	9	6	7
Model 1	1.38 (1.16–1.65)	1	0.75 (0.59–0.94)	1.16 (0.71–1.89)	3.00 (2.12–4.25)	1	0.68 (0.53–0.87)	0.73 (0.50–1.07)
Model 2	1.22 (1.01–1.47)	1	0.84 (0.66–1.07)	1.43 (0.87–2.32)	2.38 (1.61–3.53)	1	0.72 (0.55–0.93)	0.86 (0.58–1.27)
Model 3	1.15 (0.95–1.39)	1	0.85 (0.67–1.08)	1.43 (0.87–2.33)	2.11 (1.41–3.14)	1	0.73 (0.56–0.95)	0.84 (0.57–1.25)
Non-cancer non-CVD								
*N* (%)	430/5049 (8.5)	1107/16,292 (6.8)	314/4627 (6.8)	54/595 (9.1)	78/1036 (7.5)	607/13,648 (4.5)	507/12,483 (4.1)	150/3627 (4.1)
Rate/10,000 person-years	63	51	52	69	57	34	31	32
Model 1	1.23 (1.10–1.38)	1	1.01 (0.89–1.15)	1.38 (1.05–1.81)	1.65 (1.31–2.09)	1	0.93 (0.82–1.04)	0.95 (0.80–1.14)
Model 2	1.14 (1.01–1.28)	1	1.05 (0.92–1.20)	1.47 (1.11–1.96)	1.50 (1.16–1.93)	1	0.99 (0.78–1.13)	1.06 (0.87–1.29)
Model 3	1.06 (0.94–1.20)	1	1.06 (0.93–1.21)	1.44 (1.08–1.92)	1.32 (1.02–1.70)	1	1.02 (0.89–1.15)	1.06 (0.87–1.28)

Model 1: adjusted for age. Model 2: adjusted for age, ethnic background, BMI, physical activity, alcohol use, smoking status, level of education. Model 3: adjusted for age, ethnic background, BMI, physical activity, alcohol use, smoking status, level of education, non-HDL-C, hypertension, diabetes, chronic kidney disease

In the fully adjusted model, men who had >2.07 mmol/L (>80 mg/dL) HDL-C were at increased risk of all-cause mortality (HR 1.40, 95% CI 1.08, 1.67), cancer mortality (HR 1.36, 95% CI 1.05, 1.75), and non-cancer non-CVD mortality (HR 1.44, 95% CI 1.08, 1.92), but not CVD mortality, compared with men in the reference category. The rate of all-cause and cause-specific mortality was lower in women within HDL-C >2.07 mmol/L (>80 mg/dL) group compared to reference category, and women with HDL-C >2.07 mmol/L (>80 mg/dL) did not have any additional risk of mortality.

#### Non-linearity

When the results were plotted in the restricted cubic spline curves in this population, a non-linear association between HDL-C levels and all-cause mortality, cancer mortality, and non-cancer non-CVD mortality was observed for men (Fig. S4), but not for women (Fig. S5).

#### Sensitivity analyses

The associations remained statistically significant and in the same direction in the subgroup analysis (Table S6).

### HDL-C PRS

The genetic analysis included 13,007 participants from ASPREE and 62,527 participants from the UKB where genotype data and measured HDL-C levels were available. In the fully adjusted model, addition of HDL-C polygenic score and 10 PCs for population structure only slightly attenuated the association but remained statistically significant in the ASPREE cohort for all-cause mortality, cancer mortality, and “non-cancer non-CVD” mortality. However, for the UKB cohort, the associations remained unchanged (Table S7). These results indicate that common genetic variations associated with plasma HDL-C levels (represented by the HDL-C polygenic score) do not appear to contribute the identified mortality risk.

## Discussion

The principal finding of this study was a strong relationship between a very high plasma level of HDL-C and an increase in all-cause and cause-specific mortality among older generally healthy males. The strength of the observed relationships between high HDL-C levels and all-cause and other mortality in our study is notable, considering the higher frequency healthy lifestyle behaviors and a more favorable CVD risk profile among those with >2.07 mmol/L (>80 mg/dL) HDL-C concentrations. The results from the ASPREE study were further confirmed in a similar cohort derived from the UKB, providing the most comprehensive study available to date on the risks associated with very high HDL-C in this older age group.

The associations between high HDL-C levels and mortality observed in both the ASPREE and UKB cohorts were specific to men, with no evidence of similar associations in women. Although less pronounced gender difference has been reported inconsistently in other studies of a younger population [25], the difference was more prominent in the older populations studied here. The disparity may reflect a difference in sensitivity to high HDL-C levels, as noted in the large Copenhagen studies [3], which reported an increased all-cause mortality among women at the extreme upper end of the HDL-C concentration (over 3.5 mmol/L [135 mg/dL]). Similar sex differences may also exist in the lower end of HDL-C. While low levels of HDL-C are traditionally associated with increased mortality risk [26], emerging evidence suggests that high HDL-C levels might also pose a risk. Therefore, in this study, we primarily focused on examining the associations with high levels of HDL-C.

The differences between men and women may be related to differences in circulating sex hormones or result from genetic differences. In vitro studies conducted in mice [27, 28] and rats [29] have demonstrated that certain aspects of HDL-C metabolism are regulated by genes residing on the X and Y chromosomes and that dysregulation of these genes may contribute to the observed gender differences. The pathophysiological explanation for these differences has not been established.

Among men, very high HDL-C concentrations showed the strongest association with non-cancer non-CVD mortality. In ASPREE, the majority of deaths in this category were related to infections, neurological diseases, respiratory diseases, multi-organ failure, trauma, and dementia (Table S8). While an increase in cancer mortality was also observed in the ASPREE, the strength of this relationship reached statistical significance only in the UKB cohort. The relationship persisted after controlling for factors potentially related to both an increase in HDL-C and an increase in mortality. These included alcohol use, physical activity level, and chronic renal insufficiency.

Previous analyses linking very high HDL-C to mortality have been primarily conducted in populations whose mean age has been substantially younger than in our study. The findings from these studies have been inconsistent (Table S9). However, two of these studies (from Canada [2] and Norway [13]) have reported a similar pattern of cause-specific mortality. Another recent study from a younger Danish cohort reported that the relationship between a high HDL-C and all-cause mortality specifically CVD mortality was more evident in males [3].

This study adds to the existing data from older populations, building upon a previous report from the HRS study [6] that demonstrated a link between very high level of plasma HDL-C and an increased risk of all-cause mortality in healthy older adults. Unlike our study, the findings from the HRS included a U-shaped relationship between HDL-C and CVD and non-CVD mortality in both men and women [6]. Neither cancer mortality nor a sex difference was reported [6].

### CVD mortality

In both ASPREE and the UKB cohorts, the relationship between very high levels of HDL-C and CVD mortality was relatively weak and statistically non-significant in both men and women. Several previous studies, including the US-based HRS study, have reported a U-shaped relationship between HDL-C levels and CVD mortality in both men and women [3, 6]. However, most of these reports have focused on younger populations with a high preexisting CVD risk. Individuals with recognized CVD risk were excluded from both ASPREE and UKB cohorts, which may account for the lack of association we found between high HDL-C and CVD mortality.

### Cancer mortality

A higher mortality from cancer, albeit not statistically significant, had previously been reported for both men and women in both the Norwegian [13] and Danish [3] cohorts. Several studies have also reported an increased cancer risk at low HDL-C concentrations [30] indicative of a U-shaped relationship. Data from the large Norwegian cohort suggested that the relationship between high HDL-C and cancer mortality may vary across different cancer types, with the most pronounced associations observed in cancers of the respiratory, endocrine, and gastrointestinal systems [13].

### Non-cancer non-CVD mortality

Among the Norwegian cohorts, where a similar pattern was noted and the statistical power allowed a more detailed analysis, specific causes within this category included alcoholic liver disease [13]. Recent studies reporting an association between high HDL-C levels and increased risk of bone fracture [31] and dementia [32] reflecting an increasing range of disorders potentially related to very high HDL-C concentrations. The results of the ASPREE study were also similar to those from recently reported cohorts of younger community-based participants [2–5, 7–9] and others at high CVD risk [10–12], although in these cohorts an association between a high HDL-C and an increased CVD risk was reported.

As with a previous report by Liu et al. [11], we found it unlikely that the increased mortality observed was primarily driven by rare genetic variation, considering that >5% of all men in the study had HDL-C levels in the highest category. Furthermore, the associations we observed between HDL-C levels and mortality were only marginally impacted after controlling for the HDL-C polygenic score, suggesting that common genetic variation associated with HDL-C is not driving the response or relationship.

The persistence of the relationship after restricting the analyses to Australians/Caucasians, those reporting minimal exercise habits, those without a history of prostate cancer or treatment with androgen replacement (men), or treatment with hormone replacement therapy (women), and stratifying for weight loss in the previous 2 years reduces the likelihood of confounding by these factors. The likelihood that the relationship could be explained by “reverse causation” was addressed in the Danish study by Madsen et al. [3] who undertook a sensitivity analysis excluding deaths within 5 years of HDL-C measurement, again producing little change in the relationship. Although high alcohol intake is associated with increases in HDL-C levels and mortality, the results persisted among UKB participants who reported they never drink alcohol (Table S10).

## Strengths and limitations

Compared with previous reports, this study has key methodological strengths. These include its generalizability to a well-defined healthy community-dwelling population free of CVD, significant cognitive decline, and any disease that can limit survival within 5 years after enrolment. The principal limitation was the small numbers which restricted our ability to explore risks in the upper 1 or 2 percent of the distribution in women. This would have allowed a comparison with the finding by Madsen et al. [3] showing an increased mortality at these extreme levels in women. Also, the study reports association with HDL-C concentrations rather than particle size and function. Furthermore, HDL-C PRS was derived from a male-dominant GWAS (92% males in the Million Veteran Program) [23]. Further investigations are needed to explore potential heterogeneity of genetic effects between sexes on HDL-C.

Our data suggests that very high plasma HDL-C levels are associated with mortality in older men, from cancer, “non-cancer non-CVD,” and all-cause. There was no similar relationship observed among women from either cohort, suggesting the relationship is sex-specific. This data adds to the developing evidence indicating harm associated with very high HDL-C levels, predominantly affecting males, and involving an increase in cancer and other causes of increased mortality, but not CVD mortality.

### Supplementary information

ESM 1 (PDF 872 kb)

## Data Availability

The data that support the findings of this study are available from the corresponding author on reasonable request.

## Declarations

### Disclaimer

The sponsor had no role in the design and conduct of this study; collection, management, analysis, and interpretation of the data; and decision to submit the manuscript for publication but was given the opportunity to review and comment on the manuscript.
