# Supplementary material for: Sex-dependent associations of plasma high-density lipoprotein cholesterol and mortality risk in healthy older men and women: two prospective cohort studies
Source: GeroScience. 2023 Aug 23;46(2):1461–75. doi: 10.1007/s11357-023-00904-4 (PMC10828260; doi:10.1007/s11357-023-00904-4)
Supplement: Supplementary file 1 — (PDF 872 kb) [file 11357_2023_904_MOESM1_ESM.pdf]

**Supplemental Online Content**

# Association of Plasma High-Density Lipoprotein Cholesterol and Mortality in Healthy Older Adults

Table S1: Information of 309 variants used in the polygenic risk score for HDL-C.

Table S2: Association between baseline high-density lipoprotein cholesterol (HDL-C) and mortality ASPREE: Excluding the US population, participants reporting high physical activity and adjustment for weight change from baseline to annual visit 2 (HR, 95% CI)

Table S3: Association between baseline high-density lipoprotein cholesterol (HDL-C) and mortality excluding those with a history of prostate cancer and testosterone supplements ASPREE (HR, 95% CI), only males

Table S4: Association between baseline high-density lipoprotein cholesterol (HDL-C) and mortality excluding those who were taking hormone replacement therapy ASPREE (HR, 95% CI), only females

Table S5: Baseline characteristics of participants: Overall, and high-density lipoprotein cholesterol (HDL-C) category in the UK Biobank cohort

Table S6: Association between baseline HDL and mortality in the UK Biobank: Caucasians only, Excluding those reporting high physical activity (HR, 95% CI)

Table S7: Association between baseline high-density lipoprotein cholesterol (HDL-C) and mortality adjusted for HDL-C-PRS in the Aspirin in Reducing Events in the Elderly (ASPREE) and the UK Biobank (UKB) cohort (HR, 95% CI)

Table S8: Death endpoint subtype for non-cancer non-cardiovascular cause

Table S9: Comparison of associations between elevated high-density lipoprotein cholesterol (HDL-C) and cause and sex-specific mortality events observed in ASPREE, UKB and other previously published studies.

Table S10: Association between baseline high-density lipoprotein cholesterol (HDL-C) and mortality in those never drink alcohol UKB (HR, 95% CI) (n=3076, age range 40-73 years)

Fig S1. Distribution of HDL polygenic risk score: A. Aspirin in Reducing Events in the Elderly B. UK biobank

Fig S2. Distribution of HDL cholesterol concentrations in the Aspirin in Reducing Events in the Elderly (ASPREE): A. Males B. Females

Fig S3. Distribution of high-density lipoprotein cholesterol (HDL-C) concentrations in the UK Biobank (UKB): A. Males B. Females

Fig S4. Nonlinear association between high-density lipoprotein (HDL) cholesterol levels in men in the UK Biobank (UKB): A. all-cause mortality, B. Cancer mortality, C. CVD mortality and D. noncancer-non-CVD mortality

Fig S5. Nonlinear association between high-density lipoprotein (HDL) cholesterol levels in women in the UK Biobank (UKB): A. all-cause mortality, B. Cancer mortality, C. CVD mortality and D. noncancer-non-CVD mortality

## Supplementary Method: Validation cohort (UK Biobank)

Table S1: Information of 309 variants used in the polygenic risk score for HDL-C.

| Chromosome: position | SNP ID | Effect allele | Non-effect allele | Weight |
|---|---|---|---|---|
| 1:23734350 | rs1767141 | A | C | -0.0263027 |
| 1:26902388 | rs6668958 | T | G | 0.0203878 |
| 1:27236212 | rs17162330 | T | C | 0.0412983 |
| 1:28344980 | rs12144891 | A | G | 0.0167868 |
| 1:40028180 | rs4660293 | A | G | 0.04 |
| 1:63113719 | rs1168089 | T | C | -0.0153 |
| 1:93584606 | rs4847399 | A | G | -0.0216 |
| 1:107549245 | rs2878349 | A | G | -0.0149869 |
| 1:109817590 | rs12740374 | T | G | 0.0453754 |
| 1:110082886 | rs7550711 | T | C | -0.0489667 |
| 1:110163879 | rs28362581 | A | G | -0.0275937 |
| 1:110470764 | rs333947 | A | G | -0.0266884 |
| 1:150940625 | rs267738 | T | G | -0.0239485 |
| 1:156700651 | rs12145743 | T | G | -0.0139968 |
| 1:172346548 | rs1011731 | A | G | 0.0134303 |
| 1:178515312 | rs4650994 | A | G | -0.0193124 |
| 1:182157235 | rs2243976 | A | G | 0.0289208 |
| 1:205631767 | rs16856110 | A | G | 0.0199 |
| 1:219631981 | rs6694509 | T | C | -0.0194876 |
| 1:220970593 | rs2807834 | T | G | -0.0238182 |
| 1:230295691 | rs4846914 | A | G | 0.0468203 |
| 1:230416744 | rs1043900 | A | G | 0.0233713 |
| 1:234853406 | rs558971 | A | G | -0.0138399 |
| 2:272203 | rs11553746 | T | C | 0.015806 |
| 2:622827 | rs2867125 | T | C | 0.0172779 |
| 2:3634753 | rs4850047 | T | C | 0.0201487 |
| 2:21231524 | rs676210 | A | G | 0.0617334 |
| 2:21288321 | rs562338 | A | G | 0.0177805 |
| 2:53992622 | rs36020289 | C | G | 0.0462 |
| 2:65281401 | rs12990465 | T | C | 0.0263 |
| 2:165528876 | rs13389219 | T | C | 0.0327 |
| 2:203519783 | rs6435161 | T | G | 0.0177 |
| 2:227093745 | rs2943641 | T | C | 0.0391 |
| 3:11619958 | rs11712666 | A | G | -0.0140681 |
| 3:12359049 | rs2067819 | A | G | 0.0211455 |
| 3:12434901 | rs2292101 | T | C | -0.0438087 |
| 3:36979042 | rs6777217 | A | G | -0.0129 |
| 3:47045846 | rs2305637 | T | C | -0.0263536 |
| 3:50093209 | rs6762477 | A | G | 0.0232478 |
| 3:52532118 | rs13326165 | A | G | 0.0213334 |
| 3:53125922 | rs11242 | T | C | 0.0150055 |
| 3:123190731 | rs35000036 | T | C | -0.0132 |
| 3:136006576 | rs1279840 | T | C | 0.028 |

| Chromosome: position | SNP ID | Effect allele | Non-effect allele | Weight |
|---|---|---|---|---|
| 3:152171870 | rs3773910 | C | G | -0.0168785 |
| 3:156798732 | rs900399 | A | G | -0.0213473 |
| 3:185510613 | rs7633675 | T | G | 0.0133307 |
| 3:185818882 | rs4234589 | A | G | 0.0222339 |
| 4:858332 | rs11248051 | T | C | -0.019 |
| 4:26062990 | rs10019888 | A | G | 0.0243 |
| 4:69591612 | rs293429 | T | C | -0.0133 |
| 4:88064431 | rs10023050 | A | G | -0.0152213 |
| 4:89741269 | rs3822072 | A | G | -0.0214017 |
| 4:100014805 | rs2602836 | A | G | 0.012717 |
| 4:103184239 | rs112519623 | A | G | -0.0471894 |
| 4:103188709 | rs13107325 | T | C | -0.077677 |
| 4:157670537 | rs6855363 | T | C | -0.0185 |
| 5:53297611 | rs7735253 | A | G | -0.021011 |
| 5:55806751 | rs459193 | A | G | 0.0264681 |
| 5:55861786 | rs9686661 | T | C | -0.0343413 |
| 5:67714246 | rs4976033 | A | G | 0.0134382 |
| 5:74997756 | rs10057967 | T | C | -0.0205455 |
| 5:132349654 | rs4705986 | T | G | -0.0348 |
| 5:153363334 | rs390299 | A | G | -0.0145304 |
| 5:158022041 | rs2434612 | A | G | 0.0225494 |
| 5:170459675 | rs7730898 | A | G | -0.0183 |
| 6:31105413 | rs1265099 | A | G | 0.0169989 |
| 6:31526080 | rs184070214 | A | G | -0.0445304 |
| 6:31903804 | rs9332739 | C | G | -0.0290151 |
| 6:32667119 | rs3135006 | T | C | -0.0194957 |
| 6:33774394 | rs2894342 | A | C | 0.0145871 |
| 6:34194866 | rs1759645 | T | C | 0.0239333 |
| 6:34268107 | rs16885998 | T | G | 0.0505644 |
| 6:34824636 | rs11755393 | A | G | 0.0303751 |
| 6:35467891 | rs41270076 | T | C | 0.0385584 |
| 6:41987451 | rs4711698 | T | C | 0.0179812 |
| 6:42932715 | rs2274517 | T | C | 0.0155937 |
| 6:43758873 | rs6905288 | A | G | -0.0295443 |
| 6:43785255 | rs35349911 | T | C | -0.0138489 |
| 6:43806609 | rs881858 | A | G | -0.0138468 |
| 6:109246891 | rs2754820 | A | G | 0.0196214 |
| 6:109574095 | rs884366 | A | G | -0.0143481 |
| 6:116325142 | rs3756772 | T | C | 0.0128002 |
| 6:127452935 | rs2745353 | T | C | -0.0201705 |
| 6:137076010 | rs6925103 | T | C | -0.0121453 |
| 6:139839423 | rs643381 | A | C | 0.0207997 |
| 6:161006077 | rs41272114 | T | C | 0.0663301 |
| 6:161082461 | rs1652507 | T | C | 0.0460033 |
| 7:1083777 | rs1997243 | A | G | -0.0222054 |

| Chromosome: position | SNP ID | Effect allele | Non-effect allele | Weight |
|---|---|---|---|---|
| 7:6449496 | rs2303361 | T | C | -0.0237664 |
| 7:17911038 | rs10282707 | T | C | -0.0258393 |
| 7:26397239 | rs1534696 | A | C | 0.0185192 |
| 7:36170883 | rs2726070 | A | G | -0.0171845 |
| 7:50305863 | rs4917014 | T | G | -0.0138 |
| 7:72856430 | rs1178979 | T | C | -0.0335 |
| 7:129663496 | rs11556924 | T | C | 0.0126053 |
| 7:130466854 | rs972283 | A | G | 0.0275923 |
| 7:150217309 | rs3735080 | T | C | -0.0136905 |
| 7:150521026 | rs7787577 | A | G | 0.0314351 |
| 8:9183339 | rs11774381 | T | C | -0.0277809 |
| 8:9183596 | rs4841132 | A | G | -0.108003 |
| 8:10643164 | rs9657541 | T | C | -0.0246395 |
| 8:19805708 | rs1801177 | A | G | -0.119999 |
| 8:19813180 | rs264 | A | G | 0.0325237 |
| 8:19813529 | rs268 | A | G | 0.237858 |
| 8:19824492 | rs13702 | T | C | -0.0589478 |
| 8:19831977 | rs17091872 | A | G | -0.0559013 |
| 8:19854773 | rs2410622 | T | C | -0.0263574 |
| 8:19860161 | rs6983170 | T | C | 0.0932811 |
| 8:19861854 | rs6651485 | A | G | 0.0538949 |
| 8:19865175 | rs2083637 | A | G | -0.0484456 |
| 8:19889872 | rs7837677 | T | C | -0.0336313 |
| 8:19928160 | rs10106652 | A | G | 0.0306335 |
| 8:19930682 | rs34859606 | C | G | -0.0194426 |
| 8:19941145 | rs6586892 | A | C | 0.0359656 |
| 8:19955920 | rs6983999 | A | T | 0.0239463 |
| 8:71099094 | rs4512408 | T | C | 0.0255 |
| 8:106357374 | rs2957447 | A | G | 0.0138 |
| 8:116599199 | rs2293889 | T | G | -0.0304473 |
| 8:121868551 | rs4871137 | T | G | -0.0212605 |
| 8:126449406 | rs17405319 | T | C | -0.0252089 |
| 8:126484526 | rs2954026 | T | G | -0.0493033 |
| 9:15305378 | rs581080 | C | G | 0.037 |
| 9:107557315 | rs13292026 | A | G | -0.0437128 |
| 9:107562804 | rs2230808 | T | C | -0.0236144 |
| 9:107578620 | rs76881554 | A | G | -0.16407 |
| 9:107586753 | rs2066714 | T | C | -0.0514204 |
| 9:107657070 | rs3905000 | A | G | -0.0547365 |
| 9:107661150 | rs10120087 | A | C | 0.0444376 |
| 9:107665978 | rs1800978 | C | G | 0.0573747 |
| 9:107669073 | rs13284054 | T | C | 0.0541049 |
| 9:107690450 | rs1800977 | A | G | 0.0256097 |
| 9:117148430 | rs10733608 | T | G | 0.0139419 |
| 9:136155000 | rs635634 | T | C | 0.0145 |

| Chromosome: position | SNP ID | Effect allele | Non-effect allele | Weight |
|---|---|---|---|---|
| 10:8601074 | rs11255744 | T | C | -0.018197 |
| 10:17260290 | rs10904908 | A | G | -0.0117604 |
| 10:46013277 | rs970548 | A | C | -0.0245 |
| 10:94839642 | rs2068888 | A | G | 0.0205217 |
| 10:101912064 | rs2862954 | T | C | -0.0172692 |
| 10:113940329 | rs2792751 | T | C | 0.0277199 |
| 10:114048792 | rs2148489 | T | C | 0.0222912 |
| 10:115789375 | rs7076938 | T | C | 0.0172093 |
| 11:823586 | rs140201358 | C | G | 0.052292 |
| 11:2936952 | rs16928809 | A | G | -0.024642 |
| 11:13355770 | rs6486121 | T | C | -0.0151899 |
| 11:14276999 | rs2303975 | A | G | 0.0192409 |
| 11:27667202 | rs925946 | T | G | -0.0121835 |
| 11:32481177 | rs7927401 | T | C | -0.0210055 |
| 11:47258853 | rs3824866 | T | C | 0.0331259 |
| 11:47298360 | rs326214 | A | G | -0.0247953 |
| 11:47663049 | rs10838738 | A | G | 0.0192826 |
| 11:61599347 | rs61897793 | A | G | 0.0238054 |
| 11:61609750 | rs174583 | T | C | -0.0424279 |
| 11:64031241 | rs35169799 | T | C | -0.0395875 |
| 11:65561468 | rs644740 | T | C | 0.0159728 |
| 11:68703959 | rs622082 | A | G | 0.0154999 |
| 11:75455021 | rs499974 | A | C | -0.0254142 |
| 11:109995944 | rs746463 | T | C | -0.0165067 |
| 11:116611827 | rs180349 | A | T | 0.0171474 |
| 11:116633947 | rs10488698 | A | G | 0.044709 |
| 11:116648917 | rs964184 | C | G | 0.132066 |
| 11:116701354 | rs138326449 | A | G | 0.715545 |
| 11:116707044 | rs138407155 | A | T | 0.355531 |
| 11:116838130 | rs12281729 | A | C | 0.0769114 |
| 11:116896155 | rs10892063 | A | C | 0.0418259 |
| 11:116973929 | rs12269901 | C | G | 0.0267786 |
| 11:117183650 | rs593245 | T | C | 0.0146071 |
| 11:122522375 | rs7941030 | T | C | -0.022857 |
| 11:126228659 | rs4937122 | T | G | 0.0316876 |
| 12:20473758 | rs7134375 | A | C | 0.0215003 |
| 12:20591332 | rs7134150 | A | G | -0.0313866 |
| 12:26443030 | rs4963975 | A | G | -0.0197903 |
| 12:49399132 | rs1126930 | C | G | -0.0372583 |
| 12:53866619 | rs784563 | T | G | 0.0152786 |
| 12:57792580 | rs11613352 | T | C | 0.0226244 |
| 12:101873956 | rs2373459 | T | C | 0.0159672 |
| 12:109937534 | rs7298565 | A | G | 0.0279893 |
| 12:111884608 | rs3184504 | T | C | -0.024342 |
| 12:121420807 | rs1183910 | A | G | 0.0126543 |

| Chromosome: position | SNP ID | Effect allele | Non-effect allele | Weight |
|---|---|---|---|---|
| 12:122963550 | rs12369179 | T | C | -0.030961 |
| 12:123200768 | rs1798192 | T | G | -0.0193286 |
| 12:123491572 | rs940904 | A | G | -0.0226219 |
| 12:124404718 | rs12317176 | T | C | -0.0224079 |
| 12:124505444 | rs863750 | T | C | -0.0171575 |
| 12:125083696 | rs12230272 | A | G | 0.0223347 |
| 12:125261593 | rs838880 | T | C | -0.0248875 |
| 12:125283766 | rs10773105 | T | C | -0.0255327 |
| 12:125292360 | rs150728540 | A | C | 0.475574 |
| 12:125299542 | rs5891 | T | C | 0.0776071 |
| 12:125327384 | rs7306660 | A | G | -0.0285815 |
| 12:125380232 | rs7298751 | A | G | -0.0425878 |
| 13:41609047 | rs17532301 | A | G | -0.0266 |
| 14:65914867 | rs10483776 | A | G | 0.0153122 |
| 14:70783943 | rs8021180 | A | G | 0.014198 |
| 14:74250126 | rs13379043 | T | C | -0.0175699 |
| 14:105277209 | rs4983559 | A | G | -0.0252 |
| 15:41847176 | rs9944249 | T | G | -0.0132233 |
| 15:43820717 | rs55707100 | T | C | -0.0934007 |
| 15:58646332 | rs4622454 | T | C | 0.0205337 |
| 15:58674695 | rs4775041 | C | G | 0.0388498 |
| 15:58676119 | rs16940147 | A | G | 0.0498861 |
| 15:58678869 | rs117901517 | T | C | 0.0377591 |
| 15:58682690 | rs34718390 | A | G | 0.0528082 |
| 15:58683366 | rs1532085 | A | G | 0.0620128 |
| 15:58686809 | rs7165077 | T | C | -0.0273046 |
| 15:58690048 | rs6494003 | A | G | 0.0927457 |
| 15:58702941 | rs16940233 | T | C | -0.0478432 |
| 15:58721447 | rs12912415 | A | G | 0.0400102 |
| 15:58730571 | rs6494006 | T | C | 0.0436024 |
| 15:58731395 | rs17301746 | T | C | 0.087949 |
| 15:58751877 | rs936960 | T | G | 0.0524001 |
| 15:58779039 | rs1869138 | T | C | -0.0281074 |
| 15:58838010 | rs6083 | A | G | -0.016189 |
| 15:58857378 | rs17269397 | A | G | -0.0261116 |
| 15:59010962 | rs424346 | T | C | 0.0544735 |
| 15:61955338 | rs12148597 | A | G | -0.0157745 |
| 15:63414083 | rs34317102 | A | C | -0.019028 |
| 15:63970456 | rs2228510 | T | C | -0.0117381 |
| 15:90214777 | rs139271800 | A | G | -0.2482 |
| 16:985891 | rs7202647 | T | C | 0.017 |
| 16:11403893 | rs12928822 | T | C | 0.0179 |
| 16:53800954 | rs1421085 | T | C | 0.0282437 |
| 16:54410447 | rs12929759 | A | G | 0.0182545 |
| 16:55029160 | rs4238772 | A | G | 0.0285827 |

| Chromosome: position | SNP ID | Effect allele | Non-effect allele | Weight |
|---|---|---|---|---|
| 16:56883438 | rs8044753 | A | G | 0.0247609 |
| 16:56921840 | rs36049418 | A | G | 0.0655532 |
| 16:56937262 | rs11648751 | T | C | -0.0303659 |
| 16:56949168 | rs37029 | A | G | -0.0242036 |
| 16:56985139 | rs9989419 | A | G | -0.0336365 |
| 16:56985514 | rs72786786 | A | G | 0.0576565 |
| 16:56986976 | rs76315536 | T | C | 0.161449 |
| 16:56994894 | rs4783961 | A | G | 0.0260019 |
| 16:56995236 | rs1800775 | A | C | 0.0317126 |
| 16:56995935 | rs34065661 | C | G | -0.484692 |
| 16:56999258 | rs7203984 | A | C | 0.0740872 |
| 16:57005479 | rs1532624 | A | C | 0.112757 |
| 16:57005550 | rs12708974 | T | C | -0.0857298 |
| 16:57007353 | rs5883 | T | C | 0.167389 |
| 16:57007451 | rs289714 | A | G | 0.0821681 |
| 16:57015091 | rs5880 | C | G | -0.137781 |
| 16:57383759 | rs506829 | T | C | 0.0192122 |
| 16:60419220 | rs16962034 | T | G | -0.0157044 |
| 16:67714560 | rs7202185 | A | G | -0.0372704 |
| 16:67928042 | rs16942887 | A | G | 0.0742214 |
| 16:71669624 | rs1345868 | A | C | 0.0144331 |
| 16:81524274 | rs12443634 | A | C | -0.0312639 |
| 17:7462969 | rs3803800 | A | G | -0.0134 |
| 17:26695832 | rs2071379 | A | G | 0.0143 |
| 17:37715426 | rs11078915 | T | C | -0.0267118 |
| 17:37813856 | rs11869286 | C | G | 0.0238863 |
| 17:37815304 | rs11556624 | C | G | 0.0867316 |
| 17:38156712 | rs4794822 | T | C | -0.0176751 |
| 17:41926126 | rs72836561 | T | C | -0.187459 |
| 17:41942109 | rs231539 | T | C | -0.0357199 |
| 17:46022065 | rs17679445 | A | G | 0.0295385 |
| 17:47422363 | rs11652146 | A | G | -0.0171784 |
| 17:65870073 | rs12602912 | T | C | -0.0176127 |
| 17:66884879 | rs10852765 | A | G | 0.0156253 |
| 17:76395430 | rs2292642 | T | C | 0.0319034 |
| 17:76437343 | rs2289750 | A | G | -0.0282954 |
| 18:21161134 | rs1788783 | T | C | 0.015 |
| 18:47097398 | rs8093249 | A | G | 0.0336754 |
| 18:47109955 | rs77960347 | A | G | -0.250466 |
| 18:47113165 | rs117623631 | T | C | 0.343183 |
| 18:47118219 | rs3786248 | T | C | -0.0589871 |
| 18:47160953 | rs7241918 | T | G | 0.0740528 |
| 18:47176793 | rs4939886 | A | G | 0.0197515 |
| 18:47209143 | rs11660468 | T | C | 0.0253264 |
| 18:57942799 | rs9956279 | T | C | -0.0168 |

| Chromosome: position | SNP ID | Effect allele | Non-effect allele | Weight |
|---|---|---|---|---|
| 19:3414088 | rs12975319 | A | G | -0.0153429 |
| 19:7244884 | rs10408844 | T | C | 0.0180243 |
| 19:7970635 | rs4804833 | A | G | -0.0149519 |
| 19:8429323 | rs116843064 | A | G | 0.245615 |
| 19:8467235 | rs2913968 | T | C | 0.016603 |
| 19:11202306 | rs6511720 | T | G | 0.0200513 |
| 19:11303554 | rs17616661 | A | G | 0.0257687 |
| 19:11347493 | rs737337 | T | C | 0.0570415 |
| 19:32917455 | rs2111504 | A | T | 0.0166861 |
| 19:33899065 | rs731839 | A | G | 0.0177915 |
| 19:45395619 | rs2075650 | A | G | 0.0378654 |
| 19:45396973 | rs77301115 | A | G | -0.0660358 |
| 19:45412079 | rs7412 | T | C | 0.0856446 |
| 19:45414451 | rs439401 | T | C | 0.0141174 |
| 19:45422946 | rs4420638 | A | G | 0.0419678 |
| 19:45448465 | rs5167 | T | G | -0.0375886 |
| 19:46307406 | rs8111071 | A | G | 0.0278068 |
| 19:47589895 | rs2303108 | T | C | 0.01366 |
| 19:52327784 | rs3752125 | T | C | -0.0204076 |
| 19:54759361 | rs12975366 | T | C | 0.0211845 |
| 19:54792761 | rs386000 | C | G | 0.0436559 |
| 19:54837165 | rs12979085 | A | G | 0.0204138 |
| 20:17596155 | rs1132274 | A | C | -0.0159 |
| 20:32648738 | rs2268086 | A | G | 0.0143913 |
| 20:33734493 | rs1415771 | A | G | -0.0129247 |
| 20:43042364 | rs1800961 | T | C | -0.142137 |
| 20:44586023 | rs3827066 | T | C | 0.0496205 |
| 20:44598670 | rs8123864 | T | C | 0.0477456 |
| 20:45592842 | rs1211644 | T | C | -0.0177758 |
| 20:46153148 | rs11700063 | A | G | 0.0205946 |
| 20:46340596 | rs4239651 | T | C | -0.020977 |
| 20:56098733 | rs6025606 | T | C | 0.0118726 |
| 20:62196253 | rs310631 | A | C | 0.013306 |
| 20:62695931 | rs6062343 | A | G | 0.0137017 |
| 21:46271452 | rs235314 | T | C | -0.0149922 |
| 21:46901973 | rs12482088 | A | C | 0.0186627 |
| 22:21932068 | rs181362 | T | C | -0.0303378 |
| 22:29451671 | rs4823006 | A | G | -0.0129774 |
| 22:30888527 | rs17738540 | T | C | -0.0184464 |
| 22:38569006 | rs738322 | A | G | -0.0198011 |
| 22:44324727 | rs738409 | C | G | 0.0155275 |

Table S2: Association between baseline high-density lipoprotein cholesterol (HDL-C) and mortality ASPREE: Excluding the US population, participants reporting high physical activity and adjustment for weight change from baseline to annual visit 2 (HR, 95% CI)

| | HDL levels | | | | | | | |
|---|---|---|---|---|---|---|---|---|
| | <40 mg/dl <1.03 mmol/L | 40-60 mg/dl 1.03-1.55 mmol/L | 60-80 mg/dl 1.55-2.07 mmol/L | >80 mg/dl >2.07 mmol/L | <40 mg/dl <1.03 mmol/L | 40-60 mg/dl 1.03-1.55 mmol/L | 60-80 mg/dl 1.55-2.07 mmol/L | >80 mg/dl >2.07 mmol/L |
| Male | | | | | Female | | | |
| Excluding the US population (n=15121)[a] | | | | | | | | |
| All-cause | 1.03 (0.85, 1.25) | 1 | 0.98 (0.83, 1.16) | 1.48 (1.15, 1.90) | 0.89 (0.59, 1.34) | 1 | 1.02 (0.86, 1.21) | 0.95 (0.76, 1.17) |
| Cancer | 1.10 (0.84, 1.43) | 1 | 0.99 (0.77, 1.26) | 1.29 (0.88, 1.90) | 0.65 (0.34, 1.24) | 1 | 0.90 (0.69, 1.38) | 0.72 (0.52, 1.11) |
| CVD | 0.93 (0.62, 1.37) | 1 | 0.80 (0.54, 1.15) | 0.90 (0.46, 1.74) | 1.26 (0.60, 2.66) | 1 | 1.09 (0.76, 1.58) | 1.45 (0.97, 2.19) |
| noncancer-nonCVD | 1.01 (0.70, 1.45) | 1 | 1.14 (0.84, 1.54) | 2.20 (1.47, 3.29) | 1.07 (0.49, 2.34) | 1 | 1.23 (0.88, 1.70) | 0.99 (0.66, 1.48) |
| Excluding those reporting high physical activity (n=6082)[b] | | | | | | | | |
| All-cause | 1.10 (0.85, 1.42) | 1 | 1.09 (0.85, 1.39) | 2.10 (1.47, 3.01) | 1.09 (0.71, 1.67) | 1 | 0.98 (0.78, 1.23) | 0.83 (0.62, 1.11) |
| Cancer | 1.30 (0.91, 1.88) | 1 | 1.14 (0.78, 1.66) | 1.51 (0.80, 2.87) | 0.80 (0.56, 1.65) | 1 | 0.80 (0.57, 1.13) | 0.54 (0.33, 0.88) |
| CVD | 1.10 (0.66, 1.81) | 1 | 1.04 (0.62, 1.75) | 1.68 (0.74, 3.79) | 1.75 (0.84, 3.63) | 1 | 0.95 (0.61, 1.49) | 1.24 (0.73, 2.09) |
| noncancer-nonCVD | 0.83 (0.51, 1.36) | 1 | 1.05 (0.68, 1.61) | 3.05 (1.80, 5.17) | 1.04 (0.47, 2.32) | 1 | 1.31 (0.89, 1.92) | 0.97 (0.60, 1.59) |
| Additional adjustment for weight change from baseline to annual visit 2 with other confounding variables (n=16392) | | | | | | | | |
| All-cause | 1.03 (0.83, 1.27) | 1 | 0.98 (0.81, 1.20) | 1.61 (1.21, 2.13) | 1.13 (0.74, 1.72) | 1 | 1.01 (0.82, 1.23) | 1.02 (0.88, 1.28) |
| Cancer | 1.18 (0.87, 1.60) | 1 | 1.03 (0.77, 1.37) | 1.33 (0.84, 2.09) | 0.84 (0.42, 1.67) | 1 | 0.90 (0.67, 1.21) | 0.76 (0.52, 1.11) |
| CVD | 0.97 (0.63, 1.49) | 1 | 0.77 (0.50, 1.18) | 1.28 (0.67, 2.42) | 1.32 (0.59, 2.94) | 1 | 1.07 (0.71, 1.60) | 1.40 (0.98, 2.19) |

| | | | | | | | | |
|---|---|---|---|---|---|---|---|---|
| noncancer-nonCVD | 0.85 (0.56, 1.29) | 1 | 1.08 (0.76, 1.54) | 2.27 (1.45, 3.54) | 1.51 (0.74, 3.09) | 1 | 1.16 (0.79, 1.69) | 1.21 (0.75, 1.77) |

Adjusted for age, country of birth, BMI, physical activity, alcohol use, smoking status, levels of education, 100mg Aspirin, nonHDL-C, hypertension, diabetes, chronic kidney disease

[a]country of birth was not adjusted [b]physical activity was not adjusted

Table S3: Association between baseline high-density lipoprotein cholesterol (HDL-C) and mortality excluding those with a history of prostate cancer and testosterone supplements ASPREE (HR, 95% CI), only males

| | HDL levels | | | |
|---|---|---|---|---|
| | <40 mg/dl<br><1.03 mmol/L | 40-60 mg/dl<br>1.03-1.55 mmol/L | 60-80 mg/dl<br>1.55-2.07 mmol/L | >80 mg/dl<br>>2.07 mmol/L |
| All cause | 1.03 (0.84, 1.25) | 1 | 0.99 (0.82, 1.18) | 1.58 (1.22, 2.04) |
| Cancer | 1.11 (0.84, 1.47) | 1 | 0.94 (0.71, 1.33) | 1.34 (0.90, 2.02) |
| CVD | 0.86 (0.57, 1.29) | 1 | 0.84 (0.58, 1.23) | 0.84 (0.42, 1.68) |
| Non-cancer non-CVD | 1.05 (0.73, 1.51) | 1 | 1.19 (0.87, 1.63) | 2.50 (1.68, 3.72) |

adjusted for age, ethnic background, BMI, physical activity, smoking status, alcohol consumption, level of education, nonHDL-C, hypertension, diabetes, chronic kidney disease

Table S4: Association between baseline high-density lipoprotein cholesterol (HDL-C) and mortality excluding those who were taking hormone replacement therapy ASPREE (HR, 95% CI), only females

| | HDL levels | | | |
|---|---|---|---|---|
| | <40 mg/dl<br><1.03 mmol/L | 40-60 mg/dl<br>1.03-1.55 mmol/L | 60-80 mg/dl<br>1.55-2.07 mmol/L | >80 mg/dl<br>>2.07 mmol/L |
| All cause | 1.01 (0.69-1.46) | 1 | 0.99 (0.83-1.17) | 0.99 (0.80-1.22) |

| | | | | |
|---|---|---|---|---|
| Cancer | 0.81 (0.45-1.47) | 1 | 0.88 (0.68-1.12) | 0.70 (0.50-1.01) |
| CVD | 1.35 (0.67-2.76) | 1 | 1.24 (0.87-1.76) | 1.63 (1.09-2.40) |
| Non-cancer non-CVD | 1.07 (0.55-2.08) | 1 | 1.00 (0.74-1.37) | 1.03 (0.71-1.49) |

adjusted for age, ethnic background, BMI, physical activity, smoking status, alcohol consumption, level of education, nonHDL-C, hypertension, diabetes, chronic kidney disease

Table S5: Baseline characteristics of participants: Overall, and high-density lipoprotein cholesterol (HDL-C) category in the UK Biobank cohort

| | Overall | <40 mg/dl<br><1.03 mmol/L | 40-60 mg/dl<br>1.03-1.55 mmol/L | 60-80 mg/dl<br>1.55-2.07 mmol/L | >80 mg/dl<br>>2.07 mmol/L | p-value |
|---|---|---|---|---|---|---|
| *Male* | | | | | | |
| N (%) | 29,735 (100) | 5861 (19.7) | 18,114 (60.9) | 5085 (17.1) | 675 (2.3) | |
| Age, mean (SD), y | 66.9 (1.5) | 66.9 (1.5) | 66.9 (1.5) | 66.9 (1.5) | 66.9 (1.5) | 0.85 |
| Ethnic background | | | | | | 0.007 |
| Caucasian | 28594 (96.3) | 5581 (95.4) | 17,432 (96.3) | 49,34 (97.1) | 647 (95.8) | |
| Others | 1110 (3.7) | 272 (4.7) | 662 (3.7) | 148 (2.9) | 28 (4.2) | |
| Low activity (walked outside <30 minutes), n (%) | 1162 (38.8) | 2409 (43.1) | 6792 (38.6) | 1732 (34.9) | 229 (34.9) | <0.001 |
| BMI, mean (SD), $kg/m^2$ | 27.6 (3.9) | 29.2 (4.1) | 27.6 (3.7) | 25.9 (3.4) | 24.9 (3.3) | <0.001 |
| Current/former smoking, n (%) | 17,331 (58.4) | 3517 (60.1) | 10,427 (57.6) | 2975 (58.5) | 412 (61.0) | 0.004 |
| Current alcohol use, n (%) | 27,866 (93.8) | 5,218 (89.2) | 17,035 (94.2) | 4947 (97.3) | 666 (98.7) | <0.001 |
| Education, n (%) | | | | | | <0.001 |
| <12 years of schooling | 16,080 (54.1) | 3,511 (60.0) | 9801 (54.2) | 2452 (48.3) | 316 (46.8) | |
| >12 years of schooling | 13,624 (45.9) | 2342 (40.0) | 8293 (45.8) | 2630 (51.8) | 359 (53.2) | |
| Hypertension, (%) | 20,845 (70.1) | 4256 (72.6) | 12652 (69.9) | 3467 (68.2) | 470 (69.6) | <0.001 |
| Chronic kidney disease (eGFR <60ml/min)[a], (%) | 1280 (4.3) | 435 (7.4) | 704 (3.9) | 129 (2.5) | 12 (1.8) | <0.001 |
| Diabetes, n (%) | 2825 (9.5) | 1044 (17.8) | 1525 (8.4) | 216 (4.3) | 40 (5.9) | <0.001 |
| HDL-C PRS, mean (SD), z score (n=29,413) | -0.00 (1.00) | -0.55 (0.94) | 0.01 (0.94) | 0.50 (0.94) | 0.82 (0.99) | <0.001 |
| | | | | | | |
| Total Cholesterol (mg/dl) | 206.2 (42.3) | 181.9 (40.4) | 207.7 (41.0) | 224.4 (39.0) | 237.9 (38.5) | <0.001 |
| Non-HDL-C (mg/dl) | 155.8 (40.3) | 146.4 (39.7) | 158.6 (40.5) | 157.3 (38.9) | 148.4 (38.2) | <0.001 |
| *Female* | | | | | | |
| N (%) | 33,114 (100) | 1171 (3.5) | 14733 (44.5) | 13322 (40.2) | 3888 (11.7) | |
| Age, mean (SD), y | 66.9 (1.5) | 66.9 (1.5) | 66.9 (1.5) | 66.9 (1.5) | 66.9 (1.5) | 0.92 |
| Ethnic background | | | | | | <0.001 |
| Caucasian | 32,029 (96.8) | 1118 (95.7) | 14,184 (96.4) | 12,942 (97.2) | 3785 (97.4) | |

| | Overall | <40 mg/dl<br><1.03 mmol/L | 40-60 mg/dl<br>1.03-1.55 mmol/L | 60-80 mg/dl<br>1.55-2.07 mmol/L | >80 mg/dl<br>>2.07 mmol/L | p-value |
|---|---|---|---|---|---|---|
| Others | 1046 (3.2) | 52 (4.4) | 525 (3.6) | 368 (2.8) | 101 (2.6) | |
| Low activity (walked outside <30 minutes), n (%) | 13708 (43.0) | 537 (49.8) | 6359 (45.1) | 5357 (41.5) | 1455 (38.3) | <0.001 |
| BMI (kg/m2) | 27.2 (4.7) | 30.3 (5.4) | 28.5 (4.8) | 26.2 (4.1) | 24.6 (3.7) | <0.001 |
| Current/former smoking, n (%) | 13569 (41.0) | 512 (43.8) | 6009 (40.9) | 5416 (40.7) | 1632 (42.0) | 0.12 |
| Current alcohol use, n (%) | 29,134 (88.1) | 894 (76.4) | 12569 (85.5) | 12024 (90.3) | 3647 (93.9) | <0.001 |
| Education, n (%) | | | | | | <0.001 |
| <12 years of schooling | 18903 (57.2) | 792 (67.7) | 9015 (61.3) | 7238 (54.4) | 1858 (47.8) | |
| >12 years of schooling | 14172 (42.8) | 378 (32.3) | 5694 (38.7) | 6072 (45.6) | 2028 (52.2) | |
| Hypertension, (%) | 18,636 (56.3) | 640 (54.7) | 8299 (56.3) | 7490 (56.2) | 2207 (56.8) | 0.65 |
| Chronic kidney disease, (eGFR <60ml/min)[a], (%) | 1761 (5.3) | 118 (10.1) | 917 (6.2) | 577 (4.3) | 149 (3.8) | <0.001 |
| Diabetes, n (%) | 1713 (5.2) | 246 (21.0) | 1065 (7.2) | 335 (2.5) | 67 (1.7) | <0.001 |
| HDL-C PRS, mean (SD), z score (n=32700) | -0.00 (1.00) | -0.81 (0.96) | -0.28 (0.94) | 0.19 (0.94) | 0.64 (0.93) | <0.001 |
| | | | | | | |
| Total Cholesterol (mg/dl) | 234.1 (44.7) | 195.6 (44.8) | 225.0 (44.2) | 241.3 (41.4) | 255.8 (40.7) | <0.001 |
| Non-HDL-C (mg/dl) | 171.8 (42.4) | 159.2 (44.3) | 173.3 (43.6) | 172.8 (41.2) | 166.0 (40.3) | <0.001 |

[a]calculated from creatinine using CKD-EPI Creatinine Equation (2021)

Table S6: Association between baseline HDL and mortality in the UK Biobank: Caucasians only, Excluding those reporting high physical activity (HR, 95% CI)

| | HDL levels | | | | | | | |
|---|---|---|---|---|---|---|---|---|
| | <40 mg/dl<br><1.03 mmol/L | 40-60 mg/dl<br>1.03-1.55 mmol/L | 60-80 mg/dl<br>1.55-2.07 mmol/L | >80 mg/dl<br>>2.07 mmol/L | <40 mg/dl<br><1.03 mmol/L | 40-60 mg/dl<br>1.03-1.55 mmol/L | 60-80 mg/dl<br>1.55-2.07 mmol/L | >80 mg/dl<br>>2.07 mmol/L |
| Male | | | | | Female | | | |
| Caucasians only[a] | | | | | | | | |
| All-cause | 1.17 (1.09, 1.26) | 1 | 0.99 (0.91, 1.08) | 1.39 (1.17, 1.67) | 1.32 (1.12, 1.55) | 1 | 0.93 (0.86, 1.00) | 0.99 (0.88, 1.11) |
| Cancer | 1.26 (1.14, 1.40) | 1 | 0.99 (0.87, 1.11) | 1.38 (1.06, 1.78) | 1.18 (0.93, 1.49) | 1 | 0.91 (0.82, 1.00) | 0.97 (0.83, 1.14) |
| CVD | 1.15 (0.95, 1.39) | 1 | 0.84 (0.67, 1.08) | 1.39 (0.84, 2.30) | 2.13 (1.42, 3.20) | 1 | 0.74 (0.57, 0.96) | 0.85 (0.57, 1.27) |
| noncancer-nonCVD | 1.07 (0.94, 1.21) | 1 | 1.04 (0.90, 1.19) | 1.41 (1.05, 1.90) | 1.28 (0.98, 1.66) | 1 | 1.02 (0.99, 1.16) | 1.06 (0.87, 1.29) |
| Excluding those reporting high physical activity[b] | | | | | | | | |
| All-cause | 1.18 (1.06, 1.31) | 1 | 0.95 (0.93, 1.09) | 1.50 (1.13, 2.00) | 1.34 (1.08, 1.66) | 1 | 0.95 (0.85, 1.06) | 1.06 (0.89, 1.26) |
| Cancer | 1.23 (1.06, 1.44) | 1 | 0.80 (0.65, 0.98) | 1.48 (1.01, 2.21) | 1.31 (0.96, 1.78) | 1 | 0.93 (0.80, 1.08) | 1.01 (0.80, 1.27) |
| CVD | 1.25 (0.95, 1.66) | 1 | 0.84 (0.56, 1.24) | 1.41 (0.62, 3.22) | 2.03 (1.15, 3.57) | 1 | 0.78 (0.53, 1.14) | 0.97 (0.54, 1.73) |
| noncancer-nonCVD | 1.07 (0.89, 1.29) | 1 | 1.21 (0.98, 1.49) | 1.57 (0.99, 2.48) | 1.19 (0.83, 1.69) | 1 | 1.03 (0.85, 1.24) | 1.17 (0.86, 1.57) |

adjusted for age, ethnic background, BMI, physical activity, alcohol use, smoking status, level of education, nonHDL-C, hypertension, diabetes, chronic kidney disease

[a]ethnic background not adjusted [b]physical activity not adjusted

Table S7: Association between baseline high-density lipoprotein cholesterol (HDL-C) and mortality adjusted for HDL-C-PRS in the Aspirin in Reducing Events in the Elderly (ASPREE) and the UK Biobank (UKB) cohort (HR, 95% CI)

| | HDL levels | | | | | | | |
|---|---|---|---|---|---|---|---|---|
| | <40 mg/dl <1.03 mmol/L N = 1233 | 40-60 mg/dl 1.03-1.55 mmol/L N=5583 | 60-80 mg/dl 1.55-2.07 mmol/L N=4307 | >80mg dl >2.07 mmol/L N=1884 | <40 mg/dl <1.03 mmol/L N = 4,468 | 40-60 mg/dl 1.03-1.55 mmol/L N= 23,987 | 60-80 mg/dl 1.55-2.07 mmol/L N= 14,609 | >80mg dl >2.07 mmol/L N= 3,717 |
| ASPREE[a] | | | | | UKB[b] | | | |
| All cause | | | | | | | | |
| Males | 0.92 (0.73-1.17) | 1 | 1.05 (0.86-1.29) | 1.43 (1.04-1.97) | 1.13 (1.05-1.22) | 1 | 1.03 (0.95-1.12) | 1.43 (1.20-1.72) |
| Females | 1.10 (0.69-1.76) | 1 | 1.05 (0.83-1.31) | 1.03 (0.78-1.38) | 1.35 (1.15-1.58) | 1 | 0.93 (0.86-1.01) | 1.00 (0.89-1.13) |
| Cancer mortality | | | | | | | | |
| Males | 1.00 (0.72-1.40) | 1 | 1.06 (0.79-1.42) | 1.32 (0.82-2.13) | 1.24 (1.11-1.37) | 1 | 1.02 (0.90-1.14) | 1.38 (1.07-1.79) |
| Females | 0.80 (0.65-1.21) | 1 | 0.89 (0.65-1.21) | 0.73 (0.48-1.11) | 1.27 (1.01-1.60) | 1 | 0.88 (0.79-0.98) | 0.93 (0.79-1.10) |
| CVD | | | | | | | | |
| Males | 0.90 (0.56-1.44) | 1 | 0.90 (0.33-1.38) | 0.78 (0.33-1.85) | 1.14 (0.94-1.39) | 1 | 0.86 (0.67-1.10) | 1.47 (0.89-2.41) |
| Females | 1.84 (0.84-4.04) | 1 | 1.12 (0.69-1.80) | 1.73 (0.98-3.06) | 2.02 (1.34-3.05) | 1 | 0.72 (0.55-0.95) | 0.85 (0.56-1.30) |
| Non-cancer non-CVD | | | | | | | | |
| Males | 0.78 (0.47-1.29) | 1 | 1.17 (0.80-1.67) | 2.03 (1.23-3.36) | 1.01 (0.89-1.14) | 1 | 1.11 (0.97-1.28) | 1.50 (1.12-2.01) |
| Females | 1.11 (0.43-2.85) | 1 | 1.33 (0.86-2.04) | 1.18 (0.69-2.03) | 1.25 (0.97-1.63) | 1 | 1.07 (0.94-1.22) | 1.16 (0.95-1.43) |

[a]adjusted for age, country of birth, BMI, physical activity, alcohol use, smoking status, level of education, 100mg Aspirin, nonHDL-C, hypertension, diabetes, chronic kidney disease, and HDL PRS z score and principal components for population structure

[b]adjusted for age, ethnic background, BMI, physical activity, alcohol use, smoking status, level of education, nonHDL-C, hypertension, diabetes, chronic kidney disease, and HDL PRS z score

Table S8: Death endpoint subtype for non-cancer non-cardiovascular cause

| Death cause | N (%) |
|---|---|
| Blood coagulation disorder | 1 (0.1) |
| Dementia | 86 (16.0) |
| GI disease | 20 (3.7) |
| Liver disease | 11 (2.0) |
| Multi-organ failure/nonspecific/ Unascertained | 42 (7.8) |
| Neurological disease/disorder | 45 (8.4) |
| Renal disease | 15 (2.8) |
| Respiratory (excl. infective pneumonia) | 87 (16.2) |
| Sepsis/Infection (incl, infective pneumonia) | 56 (10.4) |
| Suicide | 4 (0.7) |
| Trauma | 56 (10.4) |
| Other/Unknown/No information | 115 (21.4) |

Table S9: Comparison of associations between elevated high-density lipoprotein cholesterol (HDL-C) and cause and sex-specific mortality events observed in ASPREE, UKB and other previously published studies.

| | | Males | | | | Females | | | |
|---|---|---|---|---|---|---|---|---|---|
| Study | Age | All-cause | CVD | Cancer | Other | All-cause | CVD | Cancer | Other |
| ASPREE | >65 years with a mean of 75 years | +++ | - | + | +++ | - | + | - | - |
| UK Biobank | >65 years with a mean of 67 years | +++ | + | ++ | +++ | - | - | - | - |
| CANHEART cohort[2] | 57.2 years | n/a | - | - | + | n/a | - | - | + |
| Copenhagen City Heart Study and the Copenhagen General Population Study[3] | Men: 58 (range 48–68) Women: 57 (range 47–67) | +++ | +++ | + | + | + | + | + | - |
| Health and Retirement Study[6] | >65 years of age with a mean | ++ | + | n/a | + | ++ | + | n/a | + |

| | | Males | | | | Females | | | |
|---|---|---|---|---|---|---|---|---|---|
| Study | Age | All-cause | CVD | Cancer | Other | All-cause | CVD | Cancer | Other |
| | of 74.4 years | | | | | | | | |
| Combined data from the Norwegian Counties Study (1977–87), the Age 40 Program (1985–99) and the Cohort of Norway (1994–2003).[13] | aged 20–79 years<br><br>mean age 45.5 years | ++ | - | + | ++ | ++ | - | + | ++ |

+++ HR >2 and statistically significant, ++ HR >1 and statistically significant, + borderline (near) significant, - not significant, n/a not reported

Table S10: Association between baseline high-density lipoprotein cholesterol (HDL-C) and mortality in those never drink alcohol UKB (HR, 95% CI) (n=3076, age range 40-73 years)

| | HDL levels | | | |
|---|---|---|---|---|
| | <40 mg/dl<br><1.03 mmol/L | 40-60 mg/dl<br>1.03-1.55 mmol/L | 60-80 mg/dl<br>1.55-2.07 mmol/L | >80 mg/dl<br>>2.07 mmol/L |
| All cause | | | | |
| Males | 1.08 (0.79, 1.50) | 1 | 1.18 (0.70, 2.00) | 2.94 (0.72, 12.0) |
| Females | 1.14 (0.79, 1.65) | 1 | 0.98 (0.79, 1.22) | 1.25 (0.84, 1.85) |
| Cancer | | | | |
| Males | 1.41 (0.92, 2.14) | 1 | 0.88 (0.37, 2.08) | 5.94 (1.42, 24.8) |
| Females | 1.26 (0.81, 1.96) | 1 | 0.81 (0.61, 1.08) | 1.32 (0.82, 2.10) |
| CVD | | | | |
| Males | - | 1 | - | - |
| Females | 1.26 (0.27, 5.83) | 1 | 1.39 (0.57, 3.38) | 2.02 (0.43, 9.48) |
| Non-cancer non-CVD | | | | |
| Males | - | 1 | - | - |
| Females | 0.91 (0.45, 1.83) | 1 | 1.31 (0.90, 1.90) | 0.93 (0.40, 2.18) |

adjusted for age, ethnic background, BMI, physical activity, smoking status, level of education, nonHDL-C, hypertension, diabetes, chronic kidney disease

Fig S1. Distribution of HDL polygenic risk score: A. Aspirin in Reducing Events in the Elderly B. UK biobank

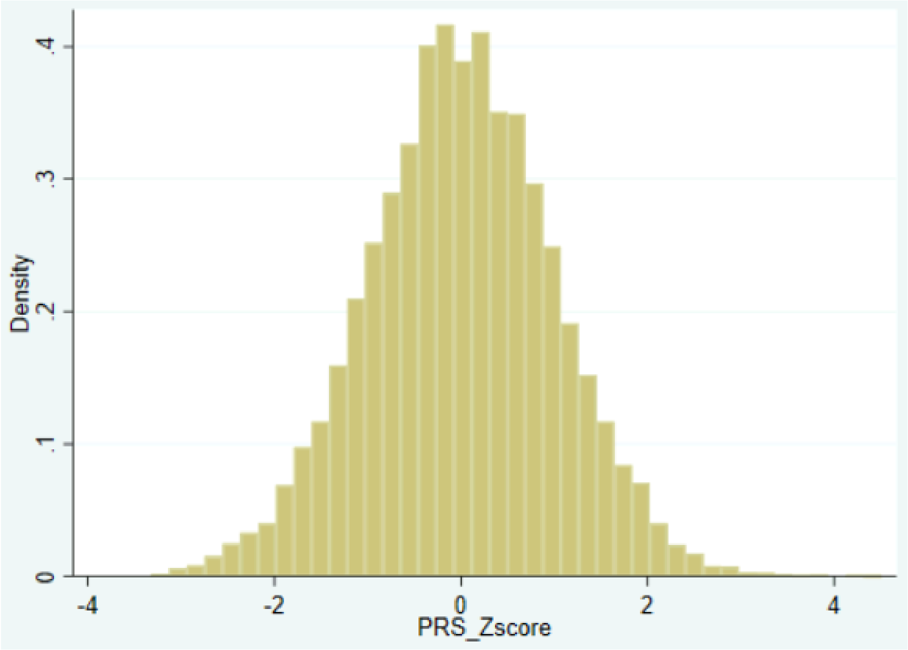


A.

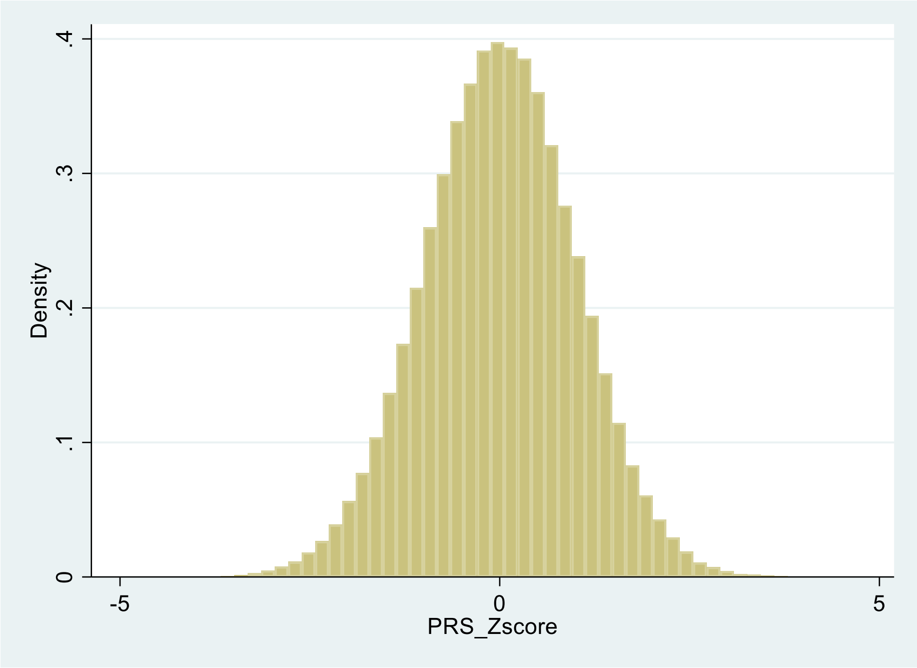


B.

Fig S2. Distribution of HDL cholesterol concentrations in the Aspirin in Reducing Events in the Elderly (ASPREE): A. Males B. Females

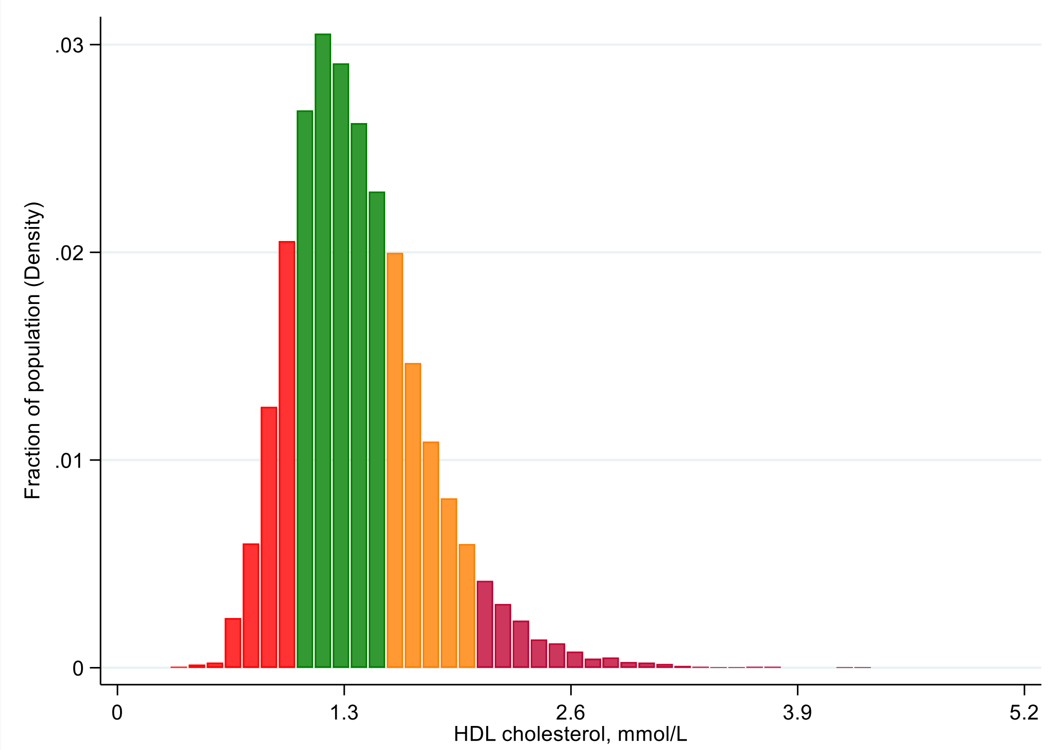


A.

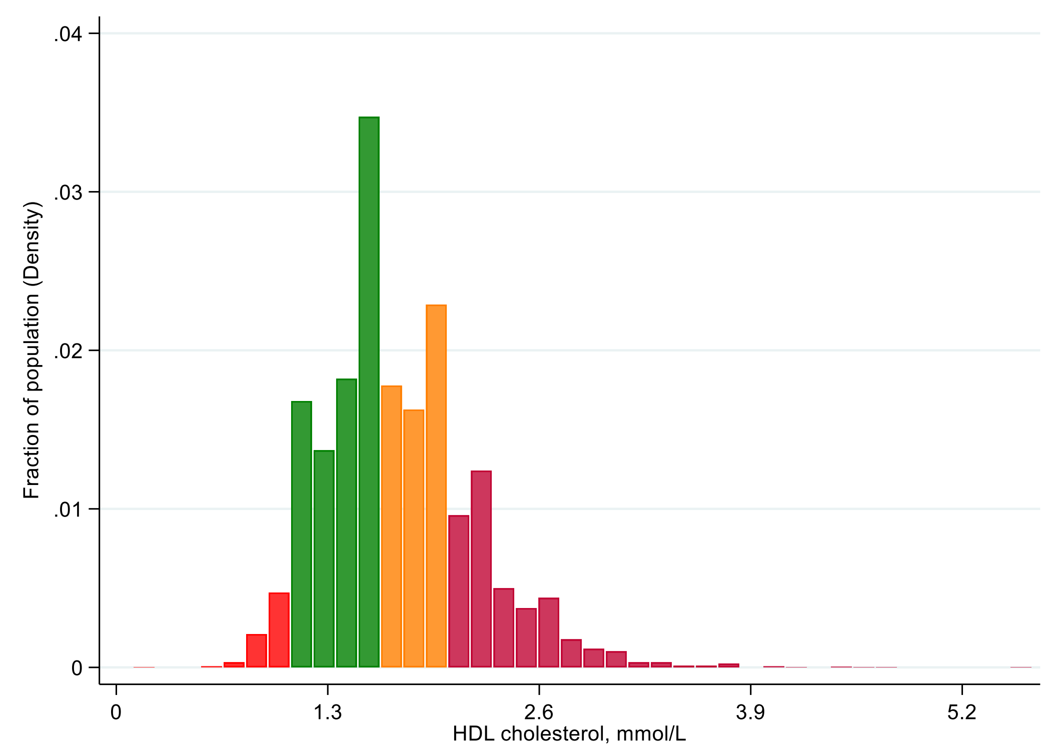


B.

Fig S3. Distribution of high-density lipoprotein cholesterol (HDL-C) concentrations in the UK Biobank (UKB): A. Males B. Females

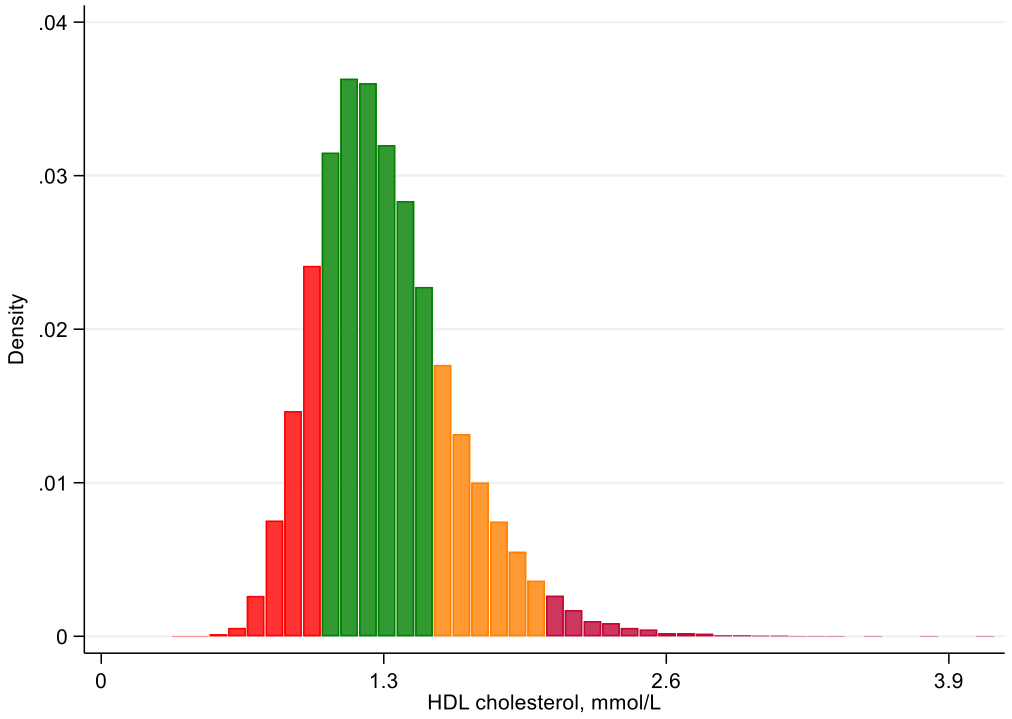


A

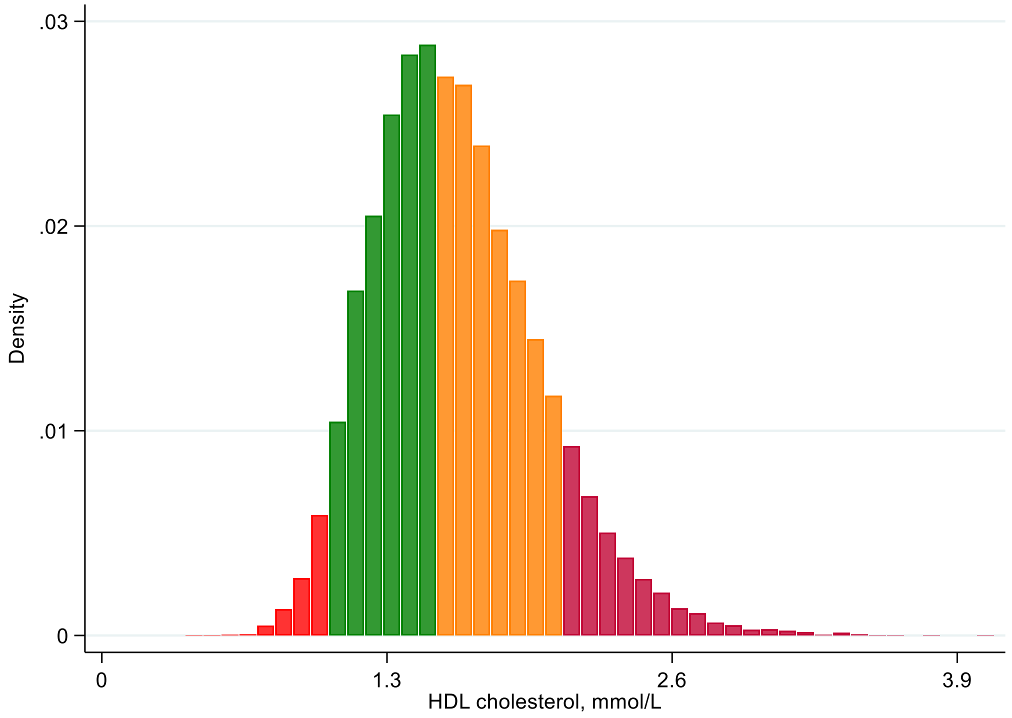


B

Figure S4. Nonlinear association between high-density lipoprotein (HDL) cholesterol levels in men in the UK Biobank (UKB): A. all-cause mortality, B. Cancer mortality, C. CVD mortality and D. noncancer-non-CVD mortality

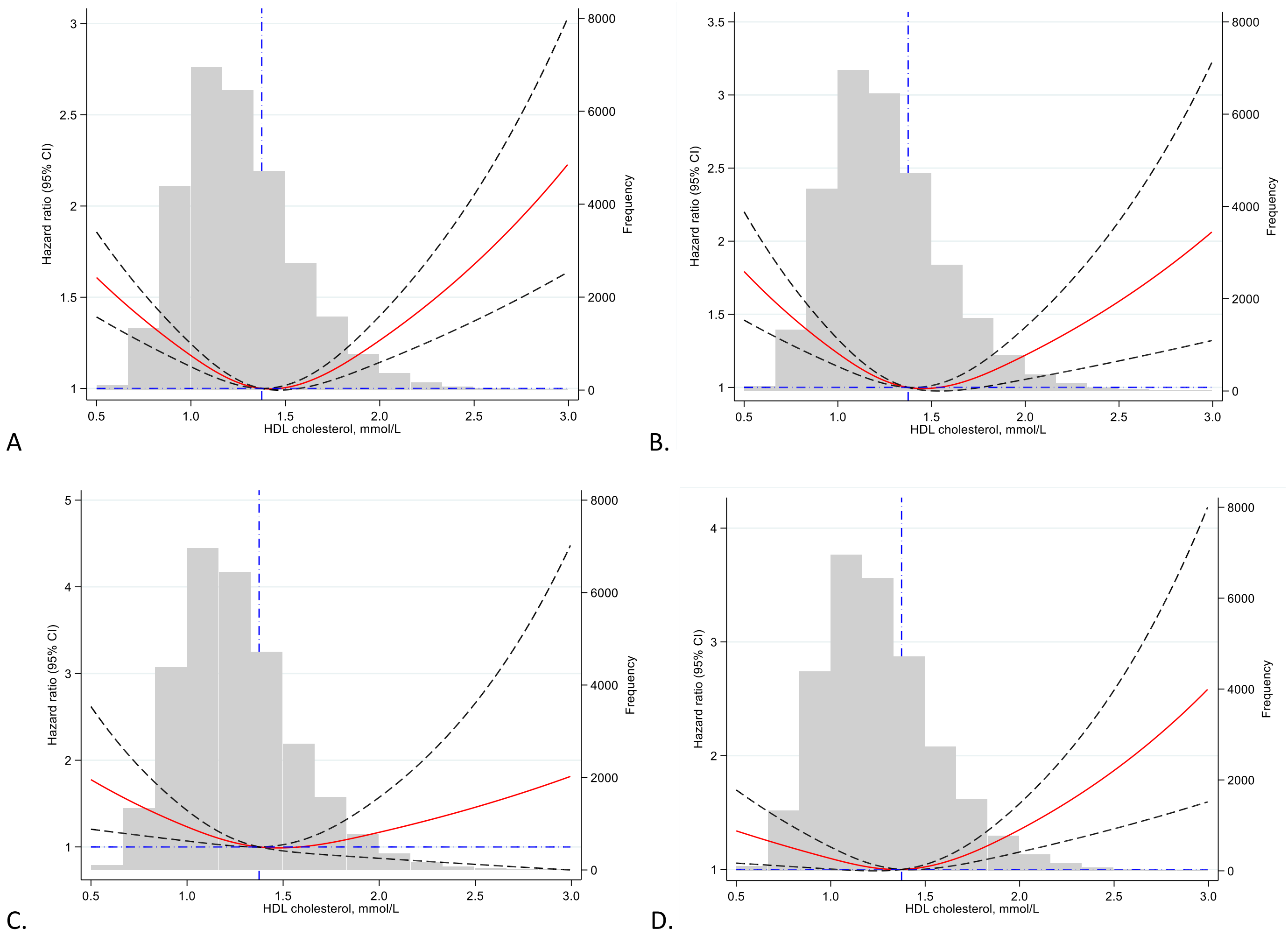

Figure S5. Nonlinear association between high-density lipoprotein (HDL) cholesterol levels in women in the UK Biobank (UKB): A. all-cause mortality, B. Cancer mortality, C. CVD mortality and D. noncancer-non-CVD mortality

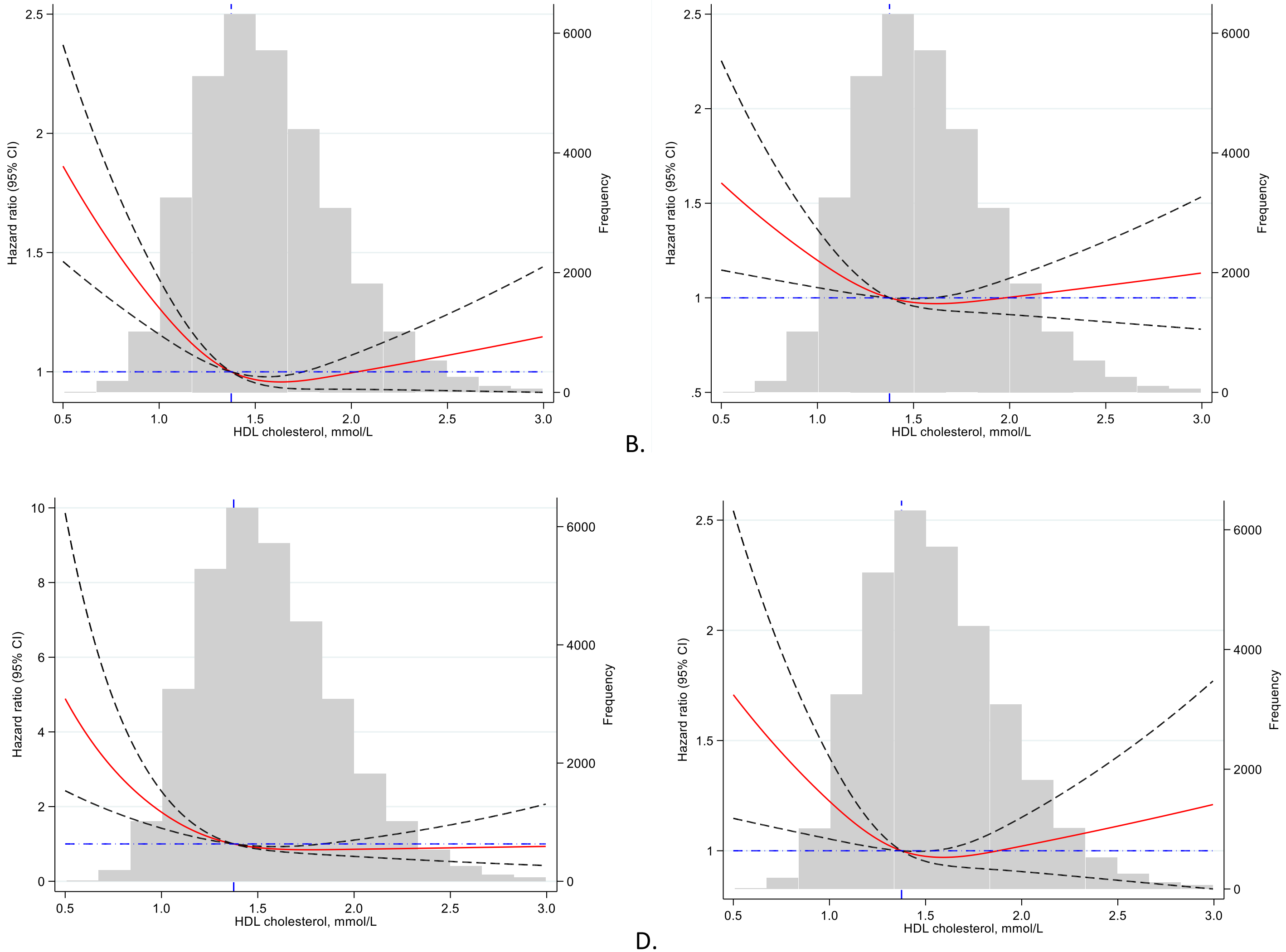

Supplementary Method: Validation cohort (UK Biobank)

**Participants exclusion criteria from UK Biobank**

We included participants at enrolment without a history of diagnosed cardiovascular events (across all hospital inpatient records, based on codes of "Diagnoses - ICD10"; UK Biobank data-field: 41270). Excluded participants were with a record of diagnosed cardiovascular events earlier than at first enrolment using ICD10:

I200, I208, I209, I210, I211, I212, I214, I219, I220, I221, I228, I229, I240, I248, I249, I251, I253, I258, I259, I460, I469, I601, I602, I603, I604, I609, I610, I611, I615, I619, I632, I633, I634, I635, I638, I639, I64, I500, I501, I509, I739, I743, I745, I440, I441, I442, I443, I446, I447, I48, I480, I481, G450, G451, G458, G459

Participants with dementia at enrolment were also excluded using ICD10:

A810, F00, F000, F001, F002, F009, F01, F010, F011, F012, F013, F018, F019, F02, F020, F021, F022, F023, F024, F028, F03, F051, F106, G30, G300, G301, G308, G309, G310, G311, G318, I673

**Identification of all-cause and cause-specific mortality from UK Biobank**

We used underlying (primary) cause of death by ICD10 (UK Biobank data-field: 40001) and contributory (secondary) causes of death by ICD10 (UK Biobank data-field: 40002) to identify cause-specific mortality.

ICD 10 codes for cardiovascular disease:

I200, I208, I209, I210, I211, I212, I214, I219, I220, I221, I228, I229, I240, I248, I249, I251, I253, I258, I259, I460, I469, I601, I602, I603, I604, I609, I610, I611, I615, I619, I632, I633, I634, I635, I638, I639, I64, I500, I501, I509, I739, I743, I745, I440, I441, I442, I443, I446, I447, I48, I480, I481, G450, G451, G458, G459

ICD 10 codes for cancer:

C00 to C97, D00 to D48

ICD 10 codes for noncancer non-CVD mortality:

Any mortality apart from the codes associated with CVD and cancer mortality were included here.
